# Emergence of Dengue 4 as Dominant Serotype During 2017 Outbreak in South India and Associated Cytokine Expression Profile

**DOI:** 10.3389/fcimb.2021.681937

**Published:** 2021-08-10

**Authors:** S. Gowri Sankar, T. Mowna Sundari, A. Alwin Prem Anand

**Affiliations:** ^1^Department of Molecular Biology, Indian Council of Medical Research (ICMR)-Vector Control Research Center - Field Station, Madurai, India; ^2^Department of Biotechnology - Bioinformatics Infrastructure Facilities (DBT-BIF) Centre (Under DBT Biotechnology Information System Network (BTISNet) Scheme), Lady Doak College, Madurai, India; ^3^Department of Biotechnology, Lady Doak College, Madurai, India; ^4^Institute of Clinical Anatomy and Cell Analysis, University of Tuebingen, Tuebingen, Germany

**Keywords:** dengue, cytokine response, primary infection, secondary infection, DENV 1-4, phylogeny, serotype

## Abstract

Dengue virus (DENV) infection is prevalent in tropical and subtropical regions of the world, which is fatal if untreated symptomatically. Emergence of new genotype within serotypes led to enhanced severity. The objective of the study is to identify the molecular characteristics of the DENV circulated during 2017 outbreak in Tamil Nadu, India, and to investigate the role of inflammatory cytokines in different “serotypes” and in “dengue severity”. A total of 135 suspected samples were tested for DENV infection using IgM, IgG, and qPCR assay; where 76 samples were positive for DENV and analyzed for 12 inflammatory cytokines using ELISA. Serotyping shows 14 DENV-1, 22 DENV-2, 7 DENV-3, and 33 DENV-4, where DENV-4 was predominant. Among 76, 42 isolates were successfully sequenced for C-prM region and grouped. A lineage shift was observed in DENV-4 genotype. Irrespective of serotypes, IFNγ was significantly elevated in all serotypes than control as well as in primary infection than secondary, indicating its role in immune response. GM-CSF and IP-10 were significantly elevated in secondary infection and could be used as prognostic biomarkers for secondary infection. Our observation shows differential cytokine expression profile varied with each serotype, indicating serotype/genotype-specific viral proteins might play a major role in dengue severity. DENV-4 as dominant serotype was reported in Tamil Nadu for the first time during an outbreak with a mixed Th1/Th17 cytokine expression profile that correlated with disease severity. We conclude it is essential to identify circulating viral genotype and their fitness by mutational analysis to correlate with disease severity and immune status, as this correlation will be helpful in diagnostics and therapeutics applications.

## Introduction

Dengue is an endemic arbovirus disease, and ~3.9 billion peoples from more than 125 countries (accounting 40% of world population) are at risk (Bhatt et al., 2013). The dengue virus has four genetically distinct serotypes (DENV 1–4) that can cause either febrile flu-like symptoms, i.e., dengue fever (DF) or severe form called dengue hemorrhagic fever (DHF) and dengue shock syndrome (DSS), which is characterized by vascular permeability and plasma leakage (Halstead and Cohen, 2015).

In India, dengue poses an increasing burden with significant underreporting (Wilder-Smith and Rupali, 2019). Two major DENV outbreaks were recorded in Tamil Nadu: (1) in 2012 with 12,826 cases and 66 deaths and GI of DENV-1 as dominant serotype (Cecilia et al., 2017) and (2) in 2017 where 23,294 cases and 65 deaths were reported (National vector-borne disease control program, Government of India). Establishment of different serotypes and emergence of new genotype within serotypes led to enhanced disease severity (Messer et al., 2003; Hapuarachchi et al., 2016), due to the exacerbation of immune response mediated by cytokines (Turner et al., 2014; Monastero and Pentyala, 2017). In DENV infection, cytokines play a vital role in disease progression and severity including thrombocytopenia, vascular permeability, and plasma leakage [reviewed in (Rothman, 2011; Srikiatkhachorn et al., 2017; Kuczera et al., 2018)], and, they can be used as diagnostic and prognostic markers [reviewed in (Srikiatkhachorn and Green, 2010; John et al., 2015; Lee et al., 2016)]. However, using cytokines/chemokines as biomarkers is a challenge due to the difference in results obtained from several studies (Lee et al., 2016). Most studies focus on dengue severity (DF, DHF, DSS) or primary and secondary infection; however, fewer reports are available on comparative study of cytokine expression within serotypes [see (Sierra et al., 2012; Cruz Hernandez et al., 2016; Yang et al., 2016)]. We hypothesize that serotype variation might induce differential cytokine expression profile among primary and secondary and with respect to severity. In this aspect, we undertook the study in the state of Tamil Nadu during the 2017 outbreak with the focus on (1) identifying molecular phylogeny of DENV circulation during the outbreak and (2) analyzing differential cytokine expression by serotypes (DENV 1–4) among primary and secondary infection, as well as in dengue severity (DF, DHF, and DSS).

## Methods

### Study Population

The study was conducted in 2017, during a dengue outbreak in the district of Madurai, Tamil Nadu, India. The study was approved by institution ethics committee of Centre for Research in Medical Entomology (CRME)-ICMR, Madurai, and all biosafety and precautionary measures were followed. Blood samples were collected in hospitals from suspected patients of dengue infection within 3–5 days on the onset of illness. For control group, blood was collected from healthy patients who were non-dengue symptomatic and checked for dengue using dengue diagnostic kit (see below). Informed written and oral consents were obtained from the patients and parents/legal guardian (in case of minors, under 18 years) who had submitted blood samples to the laboratory. The heparinated blood samples were collected and stored at −80°C until used for analysis. Dengue classification was done based on WHO classification (WHO, 1997). All experiments were performed in accordance with the relevant guidelines and regulations.

### DENV Diagnosis, Serotyping, and Sequencing

The DENV infection was diagnosed using NS1 capture ELISA kit (Panbio, Australia) as per the manufacturer’s instructions. The primary and secondary DENV infection was distinguished using IgM and IgG Capture ELISA kit (Cat. No.: E-DEN02G, Panbio, Australia). Secondary infection can be distinguished from primary infection by elevated levels of IgG and IgM : IgG ratio (Shu et al., 2003; Nguyen et al., 2018).

Serum was separated from blood, and viral RNA was extracted using Viral RNA Kit (Qiagen). DENV serotyping was done using CDC DENV-1-4 Real-Time RT-PCR (a kind gift from CDC, USA). For sequencing, the 511 bp of C-prM region was amplified using primers D1 and D2 as per methods described earlier (Lanciotti et al., 1992), and the amplified products were Sanger sequenced from both ends.

### Phylogenetic Analysis

Sequences were assembled using DNASTAR Lasergene molecular biology and submitted to ENA (European Nucleotide archive) with accession number LR595964 to LR596005 under the BioProject accession PRJEB33017. Sequences were aligned using ClustalW in MEGA7 (Kumar et al., 2016) along with reference sequences from GenBank. Based on the Tamura-Nei model with a strong bootstrap filter and 1,000 bootstrap replications (Tamura and Nei, 1993), the evolutionary relationships were analyzed using the Maximum Likelihood (ML) method. The genetic distance (p) within and between groups were calculated.

### Cytokine Assay (ELISA)

The levels of 12 inflammatory cytokines, namely, interferon-gamma (IFN-γ); interleukin (IL)-1β, 2, 4, 6, 8, 10, 12, 17A; tumor necrosis factor-alpha (TNF-α); granulocyte macrophage colony-stimulating factor (GM-CSF); and interferon gamma-inducible protein-10 (IP-10) were determined using commercial ELISA assay (Qiagen, Germany) as per the manufacturer’s instruction. Differences were considered significant when p is <0.05 or <0.01 or <0.001 or 0.0001 based on Welch’s two-tailed *t*-test.

## Results

### Clinical History

The clinical history of the patients revealed that almost all cases suffered from fever ranging from 38°C to 39.5°C. The primary infection was characterized by IgM positive samples, where IgM/IgG ratio (1:4) and IgG positive samples belong to secondary infection. Some of the most prominent symptoms in primary infection were vomiting (53.06%), myalgia (63.24%), abdominal pain (51.02%), and leukopenia (73.44%). Hypotension (77.77%) and hepatomegaly (70.37%) were dominant during secondary infection (**Table 1**). Based on WHO 1997 criteria, the DENV infection was diagnosed as DF, DHF, and DSS. Interestingly classical symptoms of DHF and DSS were observed in most primary infection cases (see **Tables 1** and **S1**).

**Table 1:** Demographic variables, serology, clinical presentations, and serotypes observed in DENV infection.

	Primary infection (N = 49)	Secondary infection (N = 27)
**Demographic variables**		
Age [Range (median)]	5–67 (39)	6–67 (13)
Gender (M:F)	24:25	16:11
Stratified age and gender [N, (%)]		
Children (5–18 years)		
Male	5 (10.2)	10 (37.03)
Female	11 (22.44)	10 (37.03)
Adult (19–67 years)		
Male	19 (38.77)	6 (22.22)
Female	14 (28.57)	1 (3.70)
**Serological assay [N, (%)]**		
NS1	29 (59.16)	17 (62.96)
IgM	36 (73.44)	11 (40.74)
IgG	0	27 (100)
**Clinical parameters [N, (%)]**		
***DF***		
1. Fever	49 (100)	27 (100)
2. Vomiting	26 (53.06)	19 (70.37)
3. Myalgia	31 (63.24)	24 (88.88)
4. Rash	7 (14.28)	12 (44.44)
5. Conjunctivitis	7 (14.28)	11 (40.74)
6. Headache	37 (75.48)	17 (62.96)
7. Leukopenia	36 (73.44)	19 (70.37)
8. Retro orbital pain	6 (12.24)	17 (62.96)
***DHF***		
1. Thrombocytopenia	4 (8.16)	26 (96.29)
2. Abdominal pain	25 (51.0)	21 (77.77)
3. Epistaxis	1 (2.04)	4 (14.81)
4. Serous effusion	1 (2.04)	22 (81.48)
5. Petechiae	21 (42.84)	21 (77.77)
6. Mucosal bleeding	4 (8.16)	17 (62.96)
7. Ascites	3 (6.12)	15 (55.55)
***DSS***		
1. Hepatomegaly	9 (18.36)	19 (70.37)
2. Hypotension	5 (10.2)	21 (77.77)
3. Shock	2 (4.08)	14 (51.85)
4. Spontaneous bleeding	1 (2.40)	8 (29.62)
5. Circulatory failure	0	6 (22.22)
Serotypes* [N, (%)]		
DENV-1	10 (20.40)	4 (14.81)
DENV-2	13 (26.52)	9 (33.33)
DENV-3	4 (8.16)	3 (11.11)
DENV-4	22 (44.88)	11 (40.74)

*Serotyping of dengue positive samples using RT-PCR.

DF, dengue fever; DHF, dengue hemorrhagic fever; DSS, dengue shock syndrome.

### Dengue Prevalence

A total of 135 samples suspected for dengue were tested using IgM, IgG, and qPCR assay, and 76 were positive for dengue infection. Among the 76 positive cases (56%), 49 belong to primary and 27 to secondary DENV infection. Serotyping of those 76 samples shows 14 DENV-1, 22 DENV-2, 7 DENV-3, and 33 DENV-4 (**Table 1**).

#### DENV Serotype Distribution

Among the 76 samples, only 42 samples were successfully sequenced for C-prM region and submitted to ENA (**Table S2**), and the phylogeny was constructed with sequences retrieved from GenBank (**Table S3**). DENV-4 was found to be predominant with 20 isolates, followed by DENV-2 with 13 isolates, and the clade was supported by 100% bootstrap value. There were only two isolates in DENV-3 supported by 100% bootstrap and seven isolates of DENV-1 with the bootstrap support of 91%. The prevalence was found to be in the following order: DENV-4 > DENV-2 > DENV-1 > DENV-3.

#### DENV Genotype Distribution

##### DENV-1

**Figure 1:**
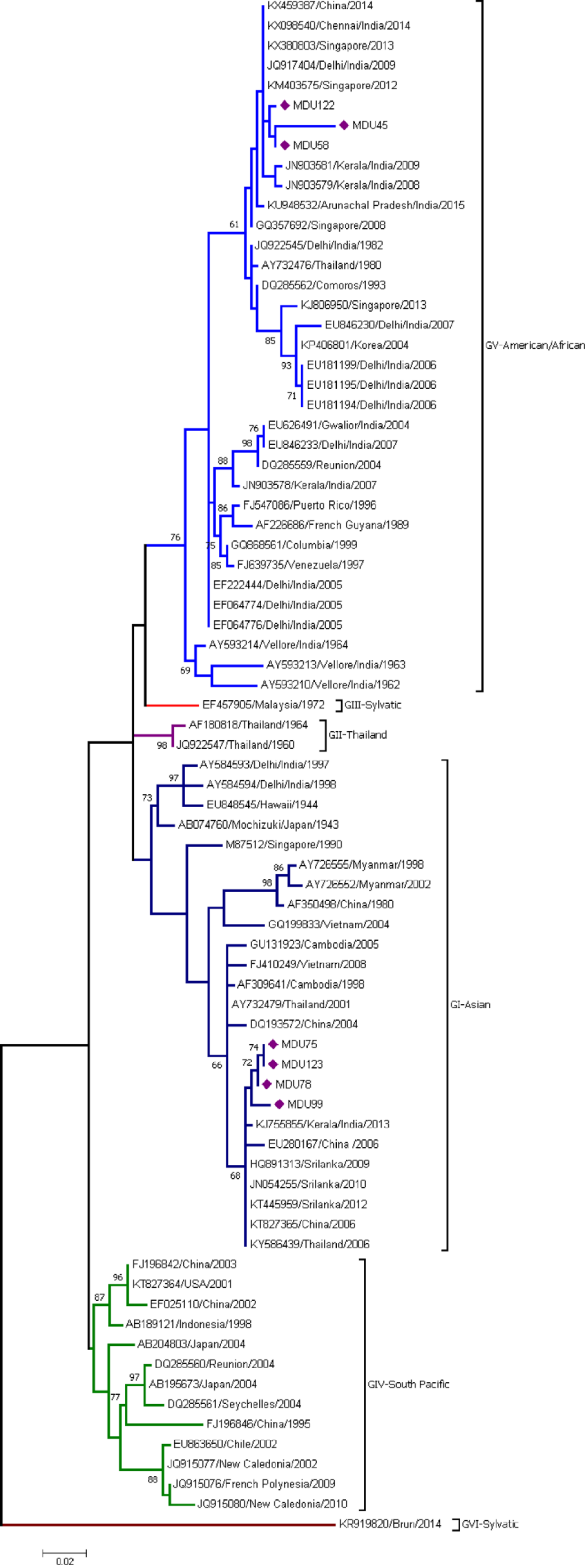
Phylogeny of DENV-1. The phylogeny of DENV-1 was inferred by using the ML method. The tree with the highest log likelihood (-2,257.26) is shown. The analysis involved 77 nucleotide sequences. There was a total of 364 positions in the final dataset. Among the seven sequences, three sequences (MDU122, MDU45, and MDU58) were grouped under genotype V (American/African), and the remaining four sequences (MDU75, MDU123, MDU78, and MDU99) were grouped with genotype I (Asian). Nucleic acid substitution per position was calculated as 0.02.

The analysis involved seven sequences from this study and 70 sequences from the database. The sequences were categorized under six genotypes (GI, GII, GIII, GIV, GV, and GVI). Three sequences—MDU122, MDU45, and MDU58—were grouped under genotype V (American/African) together with other Indian isolates from 1962 to 2014 with the supporting bootstrap value of 61%. The remaining four sequences—MDU75, MDU123, MDU78, and MDU99—were grouped with genotype I (Asian) and formed a cluster with other GI strains from Kerala, India, 2013; Sri Lanka, 2009/2010/2012; Thailand, 2006; and China, 2006, with bootstrap value of 68% (**Figure 1**). The genetic (p) distance within group was calculated as 0.025 ± 0.004 for GV-American/African and 0.034 ± 0.005 for GI-Asian (**Table 2**).

**Table 2:** The table shows p distance within group.

Serotype	Genotype	Distance within group (Mean ± SE)
DENV-1	**GI-Asian**	**0.034 ± 0.005**
	GII-Thailand	0.003 ± 0.003
	GIII-Sylvatic	n/c
	GIV-South Pacific	0.037 ± 0.005
	**GV-American/African**	**0.025 ± 0.004**
	GVI-Sylvatic	n/c
DENV-2	GI-Asian II	0.006 ± 0.004
	GII-Asian I	0.028 ± 0.007
	GIII-Asian/American	0.018 ± 0.006
	GV-American	0.043 ± 0.008
	GIVa	0.018 ± 0.004
	**GIVb**	**0.041 ± 0.005**
	Sylvatic	n/c
DENV-3	GI	0.027 ± 0.007
	GII	0.015 ± 0.004
	**GIII**	**0.019 ± 0.005**
	GV	0 ± 0
DENV-4	GI	0.012 ± 0.005
	GI Clade A	0.003 ± 0.003
	GI Clade B	0.023 ± 0.005
	**GI Clade C**	**0.008 ± 0.002**
	**GI Clade D**	**0.004 ± 0.001**
	GII	0.003 ± 0.003
	GIII	0.002 ± 0.002
	GIV (Sylvatic)	n/c
	GV	0.038 ± 0.012
	GVI	0.021 ± 0.005

The relative observations for our isolates are given in bold. p, genetic distance.

##### DENV-2

**Figure 2:**
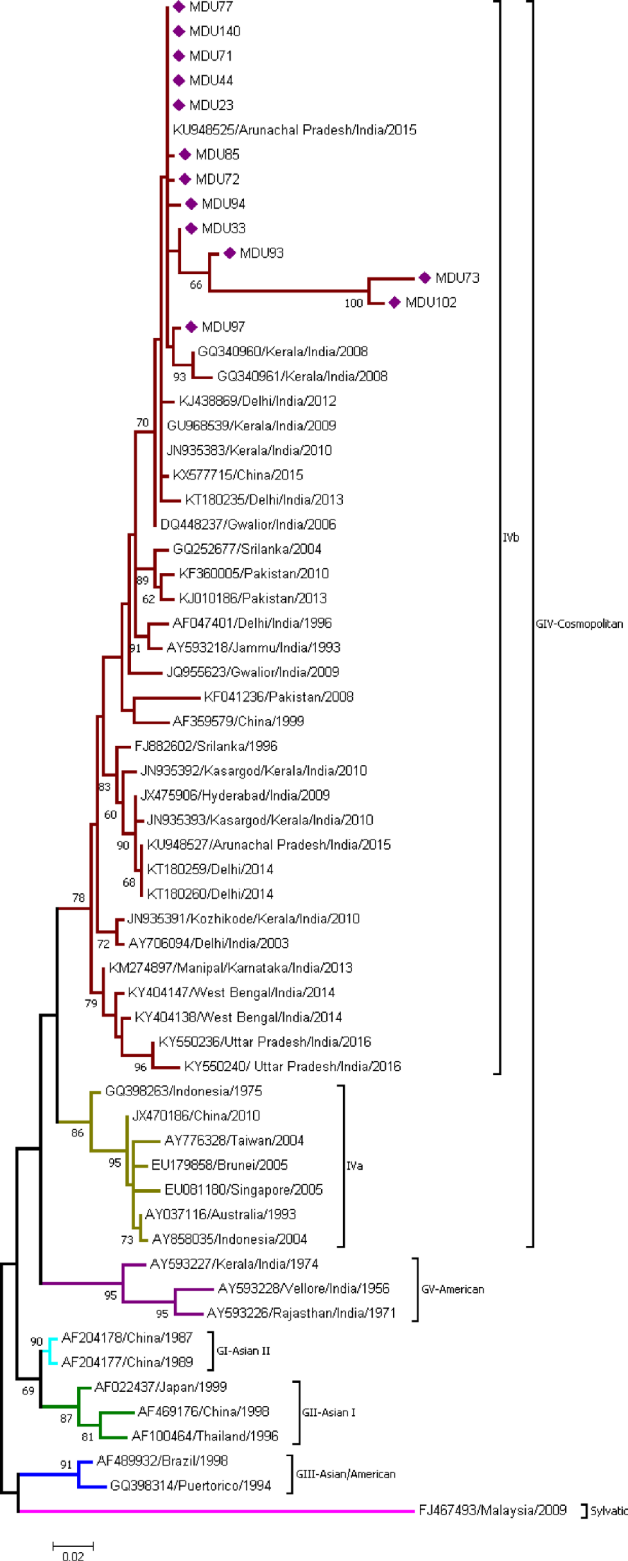
Phylogeny of DENV-2. The phylogeny of DENV-2 was inferred by using the ML method. The tree with the highest log likelihood (-2,176.94) is shown. The analysis involved 61 nucleotide sequences. There was a total of 324 positions in the final dataset. All the 13 sequences from this study were under GIV-cosmopolitan group and merged with clade B (IVb). Nucleic acid substitution per position was calculated as 0.02.

Thirteen sequences from this study and 49 sequences from the database were used to find out the monophyletic origin of DENV-2 isolates. DENV-2 sequences were categorized into five groups (GI, GII, GIII, GIV (a and b), and GV). All the isolates were under GIV-cosmopolitan group and merged with clade B (IVb) (isolates mostly from Indian subcontinent) with the mean distance 0.041 ± 0.005 (**Table 2**) and diverge from other serotypes with a range of 0.077 ± 0.012 and 0.187 ± 0.020 (**Table 3**). Among the 13 isolates, MDU102, MDU73, MDU93, and MDU33 were found to be highly similar. MDU73 and MDU102 were distant from other strains and shared 96.88% of similarity and supported with 99% bootstrap value. MDU97 was found to be similar with two strains reported from Kerala, India, 2008 (**Figure 2**).

**Table 3:** Estimates of evolutionary divergence over sequence pairs between groups.

DENV	DENV- I	DENV- II	DENV- III	DENV - IV						
**DENV-1**	–									
**DENV-2**	0.431 ± 0.284	–								
**DENV-3**	0.322 ± 0.218	0.507 ± 0.32	–							
**DENV-4**	**0.495 ± 0.358**	**0.506 ± 0.369**	**0.56 ± 0.457**	–						
**DENV-1**	**GI–Asian**	GII–Thailand	GIII Sylvatic	GIV–South Pacific	**GV–American/African**	GVI (Sylvatic)				
**GI-Asian**	–									
GII-Thailand	**0.056 ± 0.010**	–								
GIII Sylvatic	**0.074 ± 0.012**	0.047 ± 0.011	–							
GIV-South-Pacific	**0.074 ± 0.011**	0.062 ± 0.011	0.070 ± 0.011	–						
**GV-American/African**	**0.072 ± 0.011**	**0.058 ± 0.011**	**0.055 ± 0.011**	**0.064 ± 0.010**	–					
GVI Sylvatic	**0.182 ± 0.020**	0.181 ± 0.021	0.180 ± 0.021	0.173 ± 0.020	**0.195 ± 0.021**	–				
**DENV-2**	GI–Asian II	GII–Asian I	GIII–Asian/American	GIVa	**GIVb**	GV–American	GVI (Sylvatic)			
GI-Asian II	–									
GII-Asian I	0.042 ± 0.009	–								
GIII-Asian/American	0.068 ± 0.013	0.086 ± 0.014	–							
GIVa	0.064 ± 0.012	0.075 ± 0.012	0.081 ± 0.013	–						
**GIVb**	**0.077 ± 0.012**	**0.089 ± 0.012**	**0.094 ± 0.013**	**0.079 ± 0.011**	**–**					
GV-American	0.086 ± 0.013	0.090 ± 0.013	0.123 ± 0.016	0.096 ± 0.014	**0.099 ± 0.013**	–				
GVI (Sylvatic)	0.173 ± 0.020	0.177 ± 0.020	0.179 ± 0.020	0.183 ± 0.020	**0.187 ± 0.020**	0.190 ± 0.020	–			
**DENV-3**	GI	GII	**GIII**	GV						
GI	–									
GII	0.060 ± 0.015	–								
**GIII**	**0.053 ± 0.012**	**0.062 ± 0.014**	–							
GV	0.041 ± 0.011	0.048 ± 0.014	**0.051 ± 0.014**	–						
**DENV-4**	GI	GI – Clade A	GI – Clade B	**GI – Clade C**	**GI – Clade D**	GII	GIII	GIV (Sylvatic)	GV	GVI
GI	**–**									
GI - Clade A	0.038±0.011	**–**								
GI - Clade B	0.050±0.012	0.058±0.014	**–**							
**GI - Clade C**	**0.017±0.006**	**0.046±0.013**	**0.060±0.014**	**–**						
**GI - Clade D**	**0.013±0.005**	**0.042±0.012**	**0.053±0.013**	**0.019±0.007**	**–**					
GII	0.063±0.015	0.043±0.012	0.079±0.017	**0.071±0.017**	**0.069±0.016**	**–**				
GIII	0.076±0.017	0.068±0.016	0.096±0.019	**0.085±0.018**	**0.085±0.018**	0.077±0.018	**–**			
GIV (Sylvatic)	0.151±0.028	0.130±0.026	0.177±0.032	**0.162±0.030**	**0.158±0.029**	0.138±0.026	0.145±0.027	**–**		
GV	0.069±0.016	0.043±0.011	0.090±0.018	**0.068±0.016**	**0.077±0.017**	0.069±0.016	0.084±0.018	0.171±0.031	**–**	

The genetic (p) distance matrix corresponding to the DENV sequence alignment. The number of base differences per site from averaging over all sequence pairs between groups and standard error are shown.

The relative observations for our isolates in genotypes are given in bold. p, genetic distance.

##### DENV-3

**Figure 3:**
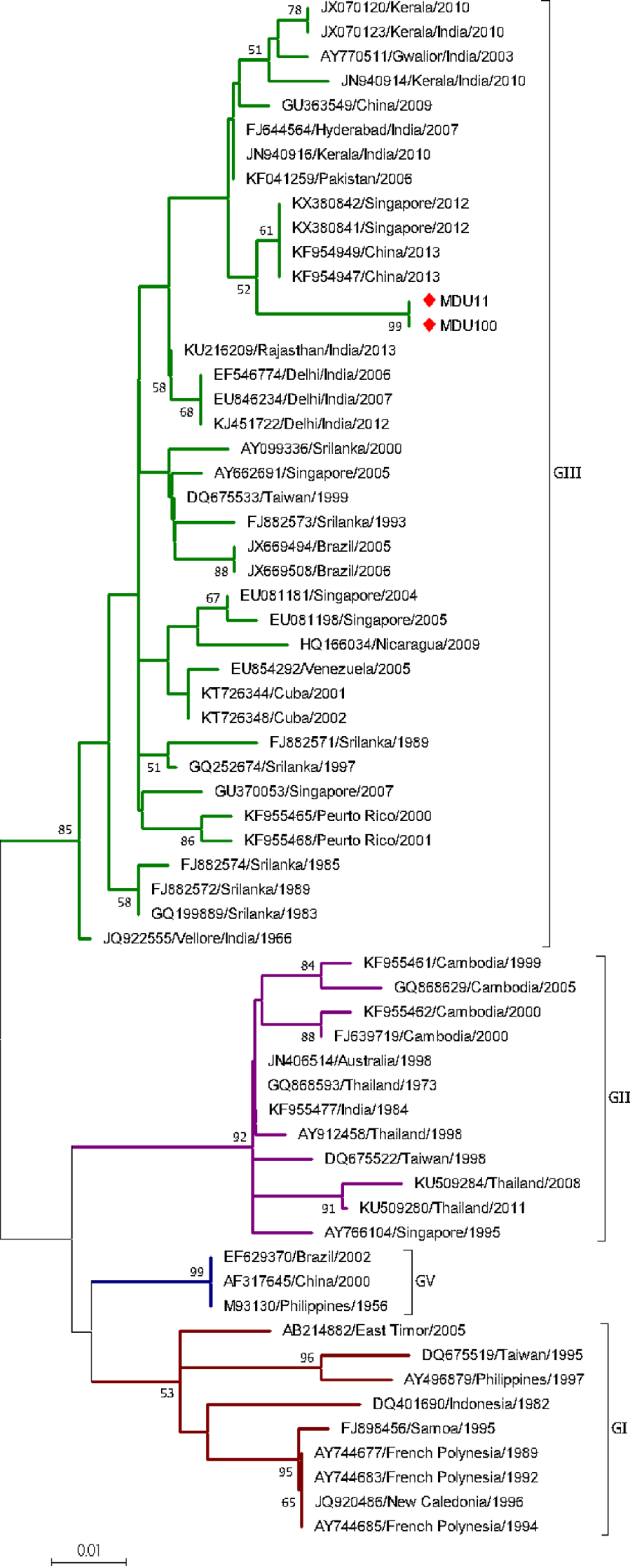
Phylogeny of DENV-3. The phylogeny of DENV-3 was inferred by using the ML method. The tree with the highest log likelihood (-1,018.14) is shown. The analysis involved 63 nucleotide sequences. There was a total of 255 positions in the final dataset. The two sequences (MDU11 and MDU100) from this study were grouped under genotype III. Nucleic acid substitution per position was calculated as 0.01.

Two sequences from the study and 61 sequences from the database were used. The sequences were categorized under four genotypes (GI, GII, GIII, and GV). Sequences MDU11 and MDU100 were grouped under genotype III with the bootstrap value 89%; mean distance within group was calculated as 0.019 ± 0.005 (**Table 2**) and diverge from other serotypes with a range of 0.051 ± 0.014 and 0.062 ± 0.014 (**Table 3**). This group contains isolates from wide geographic distribution including Caribbean, Asia, Europe, and America. Both sequences formed a separate clade with the strains reported from China (KF954949 and KF954947) and Singapore (KX380842 and KX380841) (**Figure 3**).

##### DENV-4

**Figure 4:**
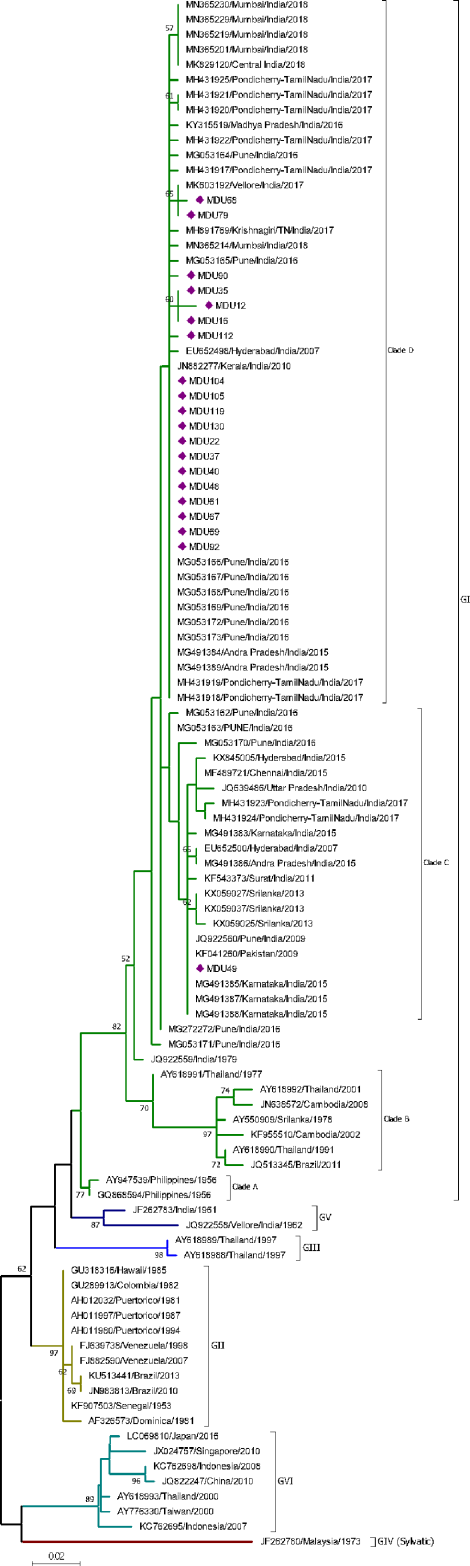
Phylogeny of DENV-4. The phylogeny of DENV-4 was inferred by using the ML method. The tree with the highest log likelihood (-1,324.25) is shown. The analysis involved 103 nucleotide sequences. There was a total of 271 positions in the final dataset. Nineteen sequences from this study were grouped under clade D of GI, and one sequence was grouped under clade C of GI. The nucleic acid substitution per position was calculated as 0.02.

The analysis of 20 nucleotide sequences from the study and 85 sequences from the database revealed that sequences of DENV-4 clustered into six genotypes (GI, GII, GIII, GIV, GV, and GVI). Genotype I was further classified as four clades (Clade A, B, C, D). All the 20 sequences were grouped under genotype I. Nineteen sequences were grouped under clade D, along with recently isolated strains from India (Pune, Mumbai, Tamil Nadu, and Pondicherry) during 2016–2018; while one isolate grouped under clade C along with other Indian strains isolated during 2009–2017 (**Figure 4**). The genetic distance within the group of Clade C and D was 0.008 ± 0.002 and 0.004 ± 0.001, respectively (**Table 2**). Though the strains of clade C and D were isolated from similar places, there exists a difference forming a separate cluster due to genetic distinction. Based on our observation and literature review, this is the first report on DENV-4 as a dominant serotype forming a genetically distinct cluster in Tamil Nadu.

### Cytokine Expression Profile

In order to understand the role of cytokines in DENV infection, 76 DENV positive samples with different serotypes (**Table 1**) were analyzed for cytokine expression.

#### Cytokine Expression In Serotypes With Respect to Primary and Secondary Infection and Disease Severity (DF, DHF, and DSS)

Several research reports show cytokines have significant differential expression during DENV infection either in primary and secondary infection or in disease severity; however, several inconsistencies were observed in the repeated studies [reviewed in (Lee et al., 2016)]. In order to understand whether serotypes with respect to primary/secondary infection and disease severity have effect on cytokine expression, we made a comparative study on differential cytokine expression: (a) between serotype DENV1–4, (b) between serotypes and primary and secondary infection, and (c) between serotypes and disease severity.

##### Comparison of Cytokine Expression in Different Serotypes

**Figure 5:**
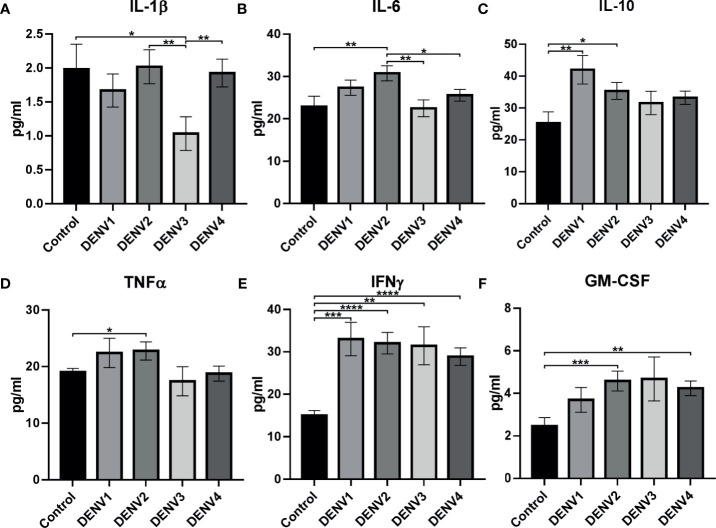
Cytokine expression in different serotypes. The graph shows statistically significant cytokine expression in different serotypes: **(A)** IL-1β, **(B)** IL-6, **(C)** IL-10, **(D)** TNFα, **(E)** IFNγ, and **(F)** GM-CSF. The statistical analysis was calculated using Welch’s two-tailed *t* test between control and different serotypes and between different serotypes. Statistical significance was shown in asterisks: *P < 0.05, **p < 0.01, ***p < 0.001, ****p < 0.0001. The error bars represent SEM. [Control (N=20), DENV1 (N=14), DENV2 (N=22), DENV3 (N=7), DENV4 (N=33)].

When cytokine expression was explored between control and different serotypes, IL-1β, IL-6, IL-10, IFNγ, TNFα, and GM-CSF show significant difference in expression (**Table S4**; **Figures 5A–F**). In DENV-1, IL-10 (*p<*0.01, **Figure 5C**) and IFNγ (*p<*0.001, **Figure 5E**) were significantly upregulated than control. In DENV-2, IL-6 (*p<*0.01, **Figure 5B**), IL-10 (*p<*0.05, **Figure 5C**), TNFα (*p<*0.05, **Figure 5D**), IFNγ (*p<*0.0001, **Figure 5E**), and GM-CSF (*p<*0.001, **Figure 5F**) were significantly upregulated than control. In DENV-3, IL-1β (*p<*0.01, **Figure 5A**) and IFNγ (*p*<0.01, **Figure 5E**) were significantly downregulated and upregulated, respectively, than control. In DENV-4, IFNγ (*p<*0.0001) and GM-CSF (*p<*0.01, **Figure 5F**) were significantly upregulated than control. Differential cytokine expression pattern was observed in serotype when compared with primary/secondary (**Figure S1**), as well as dengue severity (**Figure S2**) with respect to control.

Interestingly, within serotypes, IL-1β (**Figure 5A**) was significantly upregulated in DENV-2 (*p<*0.01) and DENV-4 (*p<*0.01) in comparison to DENV-3, while IL-6 (**Figure 5B**) was upregulated in DENV-2 in comparison to DENV-3 (*p<*0.01) and DENV-4 (*p<*0.01). This shows that cytokine expression can be prominent with respect to serotypes.

##### Comparison of Different Serotypes in Primary and Secondary Infection

**Figure 6:**
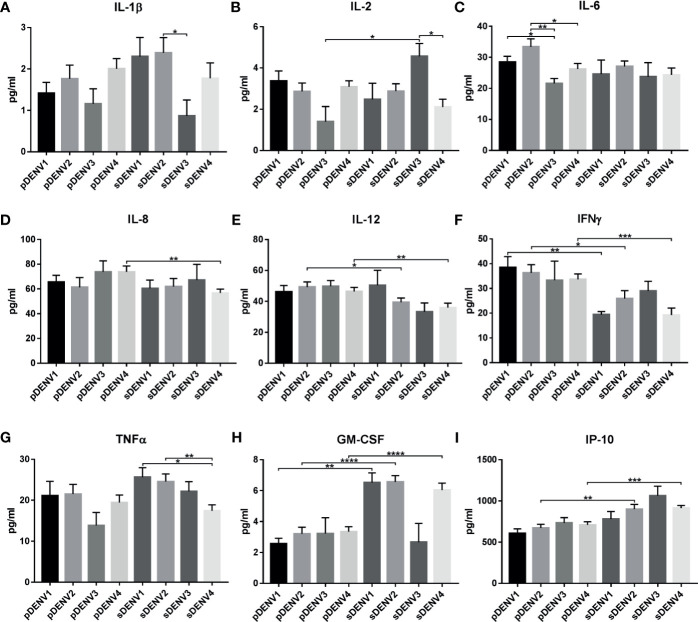
Cytokine expression in primary and secondary infection in different serotypes. The graph represents the cytokine expression within primary and secondary DENV infection. The statistically significant cytokines are **(A)** IL-1β, **(B)** IL-2, **(C)** IL-6, **(D)** IL-8, **(E)** IL-12, **(F)** IFNγ, **(G)** TNFα, **(H)** GM-CSF, and **(I)** IP-10. Statistical significance is shown in asterisk (*) mark. *P < 0.05, **p < 0.01, ***p < 0.001, ****p < 0.0001. The error bars represent SEM. [pDENV1 (N=10), pDENV2 (N=13), pDENV3 (N=4), pDENV4 (N=22), sDENV1 (N=4), sDENV2 (N=9), sDENV3 (N=3), sDENV4 (N=11)].

When comparison was made between different serotypes and primary/secondary infection, significant difference was observed in cytokines IL-1β, IL-2, IL-6, IL-8, IL-12, TNFα, IFNγ, GM-CSF, and IP-10 (**Tables S5**, **S5.2**; **Figures 6A–I**). IL-1β showed significant upregulation (*p<*0.05) in sDENV-2 than sDENV-3 (**Figure 6A**). IL-2 showed significant upregulation (*p<*0.05) in sDENV-3 than sDENV-4 (**Figure 6B**). IL-6 showed significant upregulation in pDENV-2 than pDENV-3 and 4 (*p<*0.01 and *p<*0.05 respectively), while significant downregulation (*p<*0.05) was observed in pDENV-1 than pDENV-3 (**Figure 6C**). TNFα showed significant upregulation in sDENV-1 than sDENV-4 (*p<*0.05); likewise, sDENV-2 showed similar upregulation than sDENV-4 (*p<*0.01; **Figure 6G**).

When primary and secondary infection was compared within same serotypes, significant difference in expression was observed in IL-2, IL-8, IL-12, IFNγ, GM-CSF, and IP-10 (**Tables S5**, **S5.3**; **Figures 6A–I**). In case of DENV-1, significant downregulation in IFNγ (*p<*0.01, **Figure 6F**) and upregulation in GM-CSF (*p<*0.01, **Figure 6H**) were observed in primary compared to the secondary infection. In case of DENV-2, significant upregulation in IL-12 (*p<*0.05) and IFNγ (*p<*0.05, **Figure 6H**) was observed in primary infection than secondary; while GM-CSF (*p<*0.0001, **Figure 6H**) and IP-10 (*p<*0.01, **Figure 6I**) were upregulated in secondary than primary infection. In DENV-3, only IL-2 (*p<*0.02) was significantly upregulated in secondary than primary infection (**Figure 6B**). In DENV-4, IL-2 (*p<*0.05), IL-8 (*p<*0.01), IL-12 (*p<*0.01), and IFNγ (*p<*0.001) were significantly upregulated in primary than secondary infection, while GM-CSF (*p<*0.0001) and IP-10 (*p<*0.001) were significantly upregulated in secondary infection. Taken together, cytokine expression varies with primary and secondary infection within each serotype.

##### Comparison of Serotypes and Dengue Severity (DF, DHF, and DSS)

**Figure 7:**
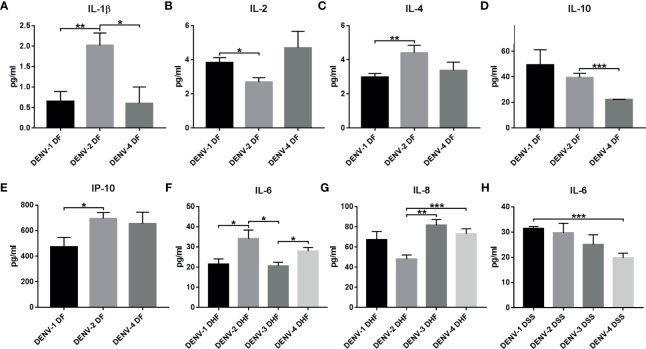
Cytokine expression within serotypes with respect to dengue severity (DF, DHF, and DSS). The graph represents the cytokine expression in dengue severity within DENV serotypes. The cytokines IL-1β **(A)**, IL-2 **(B)**, IL-4 **(C)**, IL-10 **(D)**, and IP-10 **(E)** show statistically significant expression in DF within different serotypes. IL-6 **(F)** and IL-8 **(G)** show significant expression between serotypes in DHF. IL-6 **(H)** shows significant expression within serotypes in DSS. Statistical significance is shown in asterisk (*) mark. *P < 0.05, ** p < 0.01, *** p < 0.001. The error bars represent SEM. [DF-DENV-1 (N=4), DENV-2 (N=14), DENV-4 (N=3); DHF-DENV-1 (N=5), DENV-2 (N=5), DENV-3 (N=4), DENV-4 (N=21); DSS-DENV-1 (N=5), DENV-2 (N=3), DENV-3 (N=3), DENV-4 (N=9)].

Dengue severity is one among the confounding factors in dengue disease progression. When cytokine expression was compared in DF, DHF, and DSS in different serotypes, irrespective of primary/secondary infection, significant difference of cytokine expression was observed in IL-1β, 2, 4, 6, 8, 10, and IP-10 (**Figures 7A–H**, **Tables S6**, **S6.2**). In DENV-1 significant cytokine upregulation was observed in DF where IL-2 was upregulated than DENV-2 (*p<*0.01, **Figure 7B**), and in DSS, IL-6 was upregulated than DENV-4 (*p<*0.001, **Figure 7H**). In DENV-2 significant cytokine expression was observed in DF and DHF. Within DF, DENV-2 was significantly upregulated in IL-1β (**Figure 7A**) than DENV-1 (*p<*0.01) and DENV-4 (*p<*0.01). In IL-4 (*p<*0.01, **Figure 7C**) and IP-10 (*p<*0.05, **Figure 7E**), significant increase was observed than DENV-1, and in IL-10, significant upregulation was observed than DENV-4 (*p<*0.001, **Figure 7D**). Within DHF, DENV-2 was significantly upregulated in IL-6 (**Figure 7F**) than DENV-1 (*p<*0.05) and DENV-3 (*p<*0.05). In DENV-3, difference was observed in DHF alone, where IL-8 was upregulated than DENV-2 (*p<*0.001, **Figure 7G**). In DENV-4, significant upregulation was observed in IL-8 DF than DENV-2 (*p<*0.001, **Figure 7G**). This shows that the cytokine expression varies in dengue severity with respect to different serotypes.

**Figure 8:**
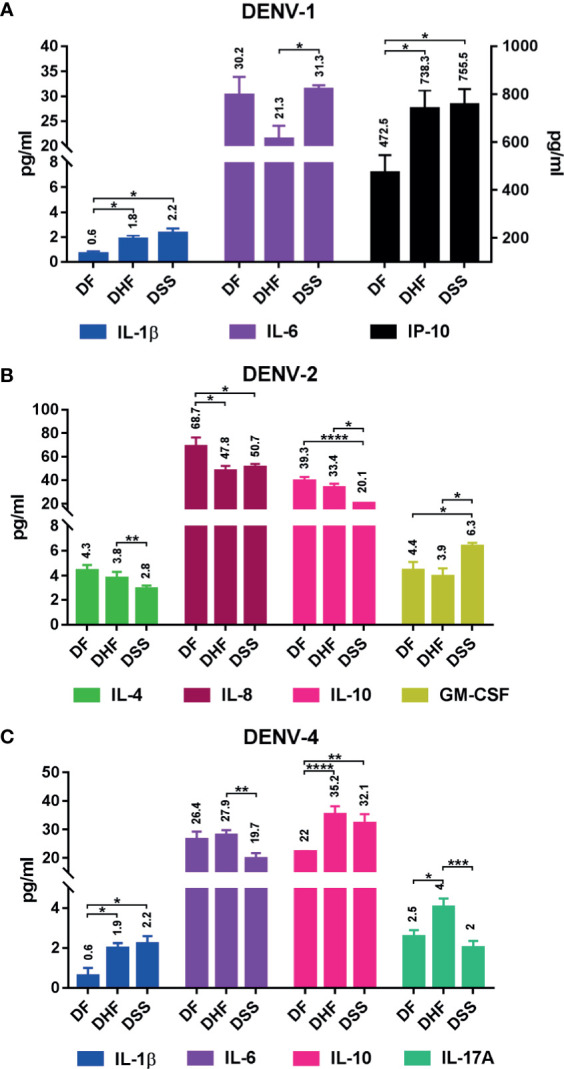
Differential cytokine expression in each serotype with respect to dengue severity. The graph shows differential expression in each serotype: **(A)** DENV-1, **(B)** DENV-2, and **(C)** DENV-4. The differential cytokine expression was analyzed using Welch’s two-tailed *t* test within dengue severity in each serotype, and significant cytokines are shown here; IL-1β, IL-6, and IP-10 show differential expression pattern in DENV-1 **(A)**; IL-4, IL-8, IL-10, and GM-CSF show differential expression pattern in DENV-2 **(B)**; and IL-1β, IL-6, IL-10, and IL-17A show differential expression pattern in DENV-4 **(C)**. Statistical significance was shown in asterisk (*): *P < 0.05, **p < 0.01, ***p < 0.001, ****p < 0.0001. The error bars represent SEM. [DF-DENV-1 (N=4), DENV-2 (N=14), DENV-4 (N=3); DHF-DENV-1 (N=5), DENV-2 (N=5), DENV-4 (N=21); DSS-DENV-1 (N=5), DENV-2 (N=3), DENV-4 (N=9)].

Differential cytokine expression pattern was observed during DF, DHF, and DSS within DENV serotypes (**Figure 8**, **Table S6.3**). In DENV-1, IL-1β had upregulated significantly in DHF (*p<*0.05) and DSS (*p<*0.05) with respect to DF. Similarly, IP-10 was also upregulated in DHF (*p<*0.05) and DSS (*p<*0.05) in comparison to DF. In severe dengue cases, IL-6 is markedly increased in DSS in comparison to DHF (*p<*0.05) (**Figure 8A**). In DENV-2, GM-CSF alone had significant upregulation in DHF (*p<*0.05) and DSS (*p<*0.05) compared to DF. Interestingly, cytokines like IL-4 (DF *vs* DSS, *p<*0.01), IL-8 (DF *vs* DHF, *p<*0.05 and DF *vs* DSS, *p<*0.05), and IL-10 (DF *vs* DHF, *p<*0.0001 and DF *vs* DSS, *p<*0.01) had significant upregulation in acute phase of infection (DF) than the DHF and DSS (**Figure 8B**). In case of DENV-4, IL-1β and IL-10 had significant upregulation in DHF (*p<*0.05 and *p<*0.0001) and DSS (*p<*0.05 and *p<*0.01), respectively, than DF. The level of IL-17A increased in DHF cases alone in comparison to DSS (*p<*0.001) and DF (*p<*0.05). IL-6 was higher in DHF than DSS (*p<*0.01) (**Figure 8C**).

## Discussion

DENV infection is an arthropod-borne viral infection with no cure. It manifests as DF and progresses to DHF/DSS, which are fatal if untreated. DHF and DSS are mostly observed during secondary heterotypic infection and in infants born to dengue-immune mothers [see review (Guzman et al., 2013; Guzman and Harris, 2015)]. In our present study, we have observed a large number of individuals with primary dengue infection had symptoms of DHF/DSS. Few other studies have also reported DHF/DSS in primary infection (Barnes and Rosen, 1974; Scott et al., 1976).

The pathophysiological mechanism behind DHF/DSS is said to be vascular permeability, which is caused by antibody-dependent enhancement (ADE), and it is postulated in secondary DENV infection (Halstead, 1979; Halstead, 2014). Experimental evidence shows antibodies against dengue proteins such as E (envelope), prM (precursor membrane), and NS1 (non-structural protein 1) promote ADE during secondary DENV infection *via* Fc receptor–bearing cells such as dendritic cells and monocytes (Dejnirattisai et al., 2010; Chotiwan et al., 2014; de Alwis et al., 2014; Halstead, 2019; St John and Rathore, 2019; Ripoll et al., 2019). However, this doesn’t answer DHF/DSS symptoms during primary DENV infection, which is observed in our study. We hypothesize that this might be due to ADE elicited by serotype/genotype-specific virulence or prior exposure to other flavivirus infection. Clapham and colleagues (2015) have shown serotype-specific primary infection in infants (<1 year old) and children aged ≥1 year old causing dengue severity DHF/DSS, where DENV-2 (Kliks et al., 1988) and DENV-4 cause disease severity in the presence of dengue antibody from maternal transfer. Another interesting factor is that DENV-3 (Kalayanarooj and Nimmannitya, 2000; Souza et al., 2007) and DENV-4 (Kalayanarooj and Nimmannitya, 2000; Rajesh et al., 2019) have been shown to be associated with serotype-specific DHF/DSS in primary infection by correlating the elevation level of liver enzymes aspartate aminotransferase (AST) and alanine aminotransferase (ALT). Japanese encephalitis virus (JEV) vaccine or prior infection has also been shown to increases DENV severity in children (Anderson et al., 2011; Jeewandara et al., 2015). In Tamil Nadu, JEV (SA 14-14-2) vaccination was carried out in children from 2014 to till date including Madurai district (Muniaraj and Rajamannar, 2019), which is the site for our study. Our observation of DHF/DSS in primary DENV infection in children (**Table S1**) might be serotype-specific ADE or due to JEV vaccination or prior JEV infection; however, further examination is needed.

Again, the above doesn’t fit with adults exhibiting DHF/DSS during primary infection. Travelers who are naïve-dengue are reported to have DHF/DSS during primary infection (Meltzer et al, 2012; Halstead and Wilder-Smith, 2019). An interesting finding by Sato and colleagues (2015) might be an answer to the above observation; they have observed DHF in a primary DENV patient who also has pre-existing JEV antibody. It also been reported that JEV pre-existing antibody (Anderson et al., 2011) or JEV vaccination (Saito et al., 2016) can facilitate ADE in DENV infection, and *in vivo* experiment shows JEV infection enhances DENV infection (Saron et al., 2018). Thus, we hypothesize that the observed DHF/DSS symptoms in primary infection in adults can be attributed to ADE facilitated by prior exposure to JEV.

### Dengue Prevalence in Tamil Nadu

In Tamil Nadu, the first dengue-like epidemic was first recorded in 1780 (Gubler, 1997). DENV-1, 2, and 4 have been first serotypically reported in Tamil Nadu from patient and mosquito sample in 1959 (Carey et al., 1966). Dengue outbreaks in Tamil Nadu of each serotype are as follows: DENV-1 dominates in 1961–62 (Carey et al., 1966; Myers et al., 1971), 2012 (Cecilia et al., 2017), DENV-2 in 1963 (Carey et al., 1966; Myers et al., 1971), DENV-3 in 1966 (Myers et al., 1968; Myers et al., 1969), and DENV-4 in 1968 (Myers et al., 1971). There are two major dengue outbreaks in Tamil Nadu, one in 2012 (Cecilia et al., 2017) and the other in 2017 (National vector-borne disease control program, Government of India). In this study, we have documented the co-circulation of all four serotypes in Madurai, Tamil Nadu during the 2017 outbreak. Though DENV-4, a rare serotype, has been isolated from various parts of India (Cecilia et al., 2011; Dash et al., 2011; Kumar et al., 2013; Shrivastava et al., 2018; Rajesh et al., 2019), ours is the first report on DENV-4 as a dominant serotype during an outbreak after 1968 (49 years) in Tamil Nadu.

### Serotype and Genotype Distribution

Genetically distinct groups within each serotype are referred as genotypes [see review (Weaver and Vasilakis, 2009)]. The serotype and genotype distributions are discussed in detail below.

#### DENV-1 Distribution

DENV-1 serotype has been further classified under five genotypes (I–V) (Weaver and Vasilakis, 2009). DENV-1 was first isolated from American soldier stationed in India from 1944 to 45 (Sabin, 1952; Snow et al., 2014) and in Tamil Nadu in 1956 (Kukreti et al., 2009).

Thirty-five sequences including three sequences from this study were clustered into genotype V (American/African) (**Figure 1**). MDU122, MDU58, and MDU45 clustered together, where MDU45 and MDU58 showed high relatedness (99.36%). The sequence MDU122, MDU58, and MDU45 showed similarity with the sequences reported from China 2014 (KX459387), Singapore 2013 and 2012 (KX380803, KM403575), Delhi 2009 (JQ917404), and Kerala 2008 and 2009 (JN903579, JN903581). The GV diverge from other genotypes GI-GVI (including sylvatic) with a range of 0.055 ± 0.011 to 0.195 ± 0.021, and within group of about 0.025 ± 0.004 (**Tables 2**, **3**).

A total of 25 sequences including four from this study formed genotype I (**Figure 1**). MDU75 is 98.96% similar with MDU78 and shows 98.33% similarity with a sequence reported from Kerala 2013 (KJ755855) and Sri Lanka 2009–2012. The genetic distance within the group GI was found to be very little, i.e., 0.034 ± 0.005 (**Table 2**), and GI diverge from other genotypes GII–GVI with a range of 0.056 ± 0.010 to 0.182 ± 0.020 (**Table 3**).

In India, circulation of DENV-1 genotype was reported to cluster in “American/African” genotype (GV, previously GIII) (Kukreti et al., 2009; Patil et al., 2011; Anoop et al., 2012; Dash et al., 2015) and in GI “Asian” genotype (Kukreti et al., 2009; Alagarasu et al., 2019). In Tamil Nadu, GI dominates only during the 2012 outbreak (Cecilia et al., 2017), but the earlier strains of DENV-1 (Vellore 1962–64, Chennai 2014) belong to GV, indicating the emergence of GI. Dual genotype circulation was reported in Vellore (Cecilia et al., 2017), Tirunelveli, Coimbatore, Dindigul, and Triuppur (Alagarasu et al., 2019) districts of Tamil Nadu; interestingly, here we report the co-circulation of Asian (GI) and American/African (GV) of DENV-1 genotype in Madurai, Tamil Nadu.

#### DENV-2 Distribution

DENV-2 serotype had been further distinguished into five major genotypes (Weaver and Vasilakis, 2009). The first DENV-2 isolate reported in India was from Vellore, Tamil Nadu during the 1956 outbreak, which is genotype V (American) (Kumar et al., 2010). DENV-2 isolates from the study were classified under GIV Cosmopolitan, as they are closer to Cosmopolitan [Indonesian 1975 GQ398263 (Christenbury et al., 2010; El-Kafrawy et al., 2016; Ahamed et al., 2019)] strain. All 13 DENV-2 sequences (**Figure 2**) showed high similarity with the sequences reported from Arunachal Pradesh 2015 (KU948525), Kerala 2008–2010 (GQ340961, GQ340960, GU968539, JN935383), Delhi 2012–2013 (KJ438869, KT180235), and Gwalior 2006 (DQ448237), supported with a bootstrap of 70, and the mean distance within group was found to be 0.041 ± 0.005, indicating these strains might be originated from these regions (**Table 2**). As per reports, the Kerala and Arunachal Pradesh strains were genetically related to circulating strains in North India, particularly Delhi and Gwalior (Anoop et al., 2010; Kumar et al., 2013; Ahamed et al., 2019), which was in circulation (Sharma et al., 2016) for more than a decade.

Global evolutionary history shows the DENV-2 transmission routes are from South America to the Caribbean and East and South Asia to Puerto Rico (Wei and Li, 2017). In India, several studies (Kumar et al., 2010; Dash et al., 2013; Kasirajan et al., 2019) show that strains before 1971 (1956–1971) belong to the American genotype, which has been replaced by Cosmopolitan genotype (i.e., 1971–till date). Taken together, our isolates show the establishment of Cosmopolitan genotype, and further, the DENV-2 isolates were introduced from North India to Tamil Nadu *via* Andhra Pradesh and Kerala.

#### DENV-3 Distribution

DENV-3 was genetically distinct into five genotypes (GI-GV) (Wittke et al., 2002). DENV-3 is first reported in Tamil Nadu/India at Vellore in 1966 (Myers et al., 1969). Though DENV-3 was circulating in India, this serotype dominated only in few outbreaks, during 2003–04 in Northern India (Dash et al., 2006) and in Pondicherry (Hoti et al., 2006). Most of the DENV-3 isolates from India during 1966–2017 (Dash et al., 2006; Hoti et al., 2006; Patil et al., 2012; Manakkadan et al., 2013; Cecilia et al., 2017; Rajesh et al., 2019; Parveen et al., 2019) fall under GIII. According to Lanciotti and colleagues (1994), the GIII subtype originated from India (Vellore 1966) and spread to other parts of the world, which is further confirmed from our phylogenetic analysis. The Vellore 1966 (JQ922555) isolate was the parent/ancestor strain for all other existing GIII strains with a bootstrap value of 85 (**Figure 3**). Interestingly, our isolates (MDU11 and MDU100) are not closely related to any of the other Indian isolates including recently isolated strains (JX070120, JX070123) from Padmanabhapuram, Tamil Nadu (Manakkadan et al., 2013); however, they are distantly related to strains of China 2013 (KF954947, KF954949) and Singapore 2012 (KX380841, KX380842) with a bootstrap value of 56, indicating these isolates might be from other Asian countries, which might be a reason for its low prevalence.

#### DENV-4 Distribution

DENV-4 is genetically distinguished into five genotypes (GI–GV) including sylvatic genotype (GIV) (Gallichotte et al., 2018); later a sixth genotype (GVI) was proposed due to the formation of separate cluster among GII strains, which includes strains from East and Southeast Asian countries (Shrivastava et al., 2018). DENV-4 was first reported in Vellore, Tamil Nadu/India in 1960 with an outbreak in 1968 (Myers et al., 1971). These Vellore strains (JQ922558 and JF262783) form a distinct group and belong to GV (Ahamed et al., 2019) [previously GI (Waman et al., 2016)]. This is the first report on DENV-4 as a dominant serotype during an outbreak in Tamil Nadu, since 1968 (after 49 years).

Interestingly, a strain (JQ922559, 1979) with an unknown place of origin in India is the ancestor for the DENV-4 GI genotype. This strain bifurcates into two distinct strains (MG053171 and MG272272) where one (MG272272) is the ancestor for all the currently existing DENV-4 strains from India including Clade C and D (**Figure 4**). From this study, MDU49 from Clade C was closer to the strain from Pune 2009 (JQ922560); the rest of the isolates falls into new Clade D [as reported by Shrivastava and colleagues (2018)] and closer to strains of DENV-4 Pune 2016.

The Clade C strains diverge from other genotypes GI-GVI with a range of 0.017±0.006 to 0.162±0.030, and within group of about 0.008 ± 0.002 (**Tables 2** and **3**). Our report is similar to that of Uehara and colleagues (2017), where the DENV-4 strains (KX059025, KX059027, and KX059037) from 2012–13 Sri Lanka epidemic, and MDU49 was closer to Pune 2009 (JQ922560) (**Figure 4**). This shows that MDU49 was from the lineage of Indian circulating DENV-4 strains.

The Clade D strains diverge from other genotype GI-GVI (including sylvatic) with a range of 0.013±0.005 to 0.158±0.029, and within group of about 0.004 ± 0.001 (**Tables 2**, **3**). Majority of the strains isolated from various parts of India from 2007 to 2016 fall into Clade C (Cecilia et al., 2011; Kumar et al., 2013; Ahamed et al., 2019; Kasirajan et al., 2019); and the strains isolated in 2017 and after (this study) in India seem to fall under the Clade D, which is an evidence for viral fitness of these strains; however, the diversity of selection should be studied in context to it.

In our phylogenetic analysis, the earliest strains are isolated in 2007 from Hyderabad [EU652500 (Clade C), EU652498 (Clade D)] (Neeraja et al., 2013); however, the parental/ancestor strains (MG053171 and MG272272) for both Clade C and D are from the 2016 outbreak in Pune. This indicates the parental strains are in existence without any prominent outbreaks. In Tamil Nadu till 2015, strains of DENV-4 fall under Clade C of GI (Kasirajan et al., 2019), and in this study, the strains isolated in 2017 and after fall into Clade D of GI. Taken together, there is a shift in Clade C to D of DENV-4 that might be due to selection pressure. Though nucleotide variation was observed, mutation among Indian DENV-4 strains was non-significant (data not shown). Further studies are needed to understand viral fitness of these strains.

### Cytokine Expression in DENV Serotypes

In dengue infection, cytokines and immune mediators, namely, several IL (1β, 2, 6, 8, 10, 12), TNFα, IFNγ, GM-CSF, and IP-10, have been reported to have significant impact [see review (Chaturvedi et al., 2000; Leong et al., 2007; Tsai et al., 2013; Wu et al., 2013; Fallahi and Elia, 2016; Lee et al., 2016; Kuczera et al., 2018)]. Cruz Hernandez and colleagues (2016) have reported IL-6, IL-12p70, and TNFα were significantly increased in DENV-2 than DENV-1, indicating different serotypes induce diverse cytokine expression. Sierra and colleagues (2012) have shown experimental evidence for variance in cytokine expression to different serotypes. In primary infection, TNFα was high in DENV-1, DENV-2, and DENV-3; whereas heterotypic (secondary) infection by DENV-2 on DENV-1 and DENV-3 persons shows high TNFα expression. Similarly, IFNγ was higher in DENV-1, 2, and 3 primary infections; however, heterotypic infection by DENV-3 significantly increased DENV-2 IFNγ expression than other serotypes (Sierra et al., 2012). Similarly in our study, we observed significant variation in cytokine expression in different serotypes and with respect to primary/secondary infection as well as dengue severity (**Figures 5**–**7**).

#### IL-10 Expression in DENV Serotypes

IL-10 is an anti-inflammatory cytokine that plays a crucial role in viral replication, persistence, and clearance (Brooks et al., 2006). Experimental dengue viral infection shows increase of IL-10 facilitates dengue replication (Ubol et al., 2010; Tsai et al., 2014). Our observation shows significant increase of IL-10 in DENV-1 and 2 (**Figure 5C**) as well as in pDENV-1, 2, and 4 than control (**Figure S1** and **Table S5.1**), which can be attributed to viral replication.

Increased IL-10 is a hallmark for DHF/DSS [see review (Tsai et al., 2013)]. Similar observation was made in DENV-4 DHF in comparison to control (**Table S6.1 ** and **Figure S2**). Interestingly, IL-10 was also significantly elevated in DENV-2 DF in comparison to control (**Table S6.1**, **Figure S2**) and DENV-4 DF (**Figure 7D**), which contradicts most studies where IL-10 was increased in DHF/DSS. Research shows that IL-10 is predominantly associated with monocytes with significant increase contributing to DHF/DSS (Ubol et al., 2010; Malavige et al., 2013). There were researches that contradict IL-10’s role in DHF/DSS (Boonnak et al., 2008; Kou et al., 2011); where most of the studies have been conducted with heterotypic infection. Sierra and colleagues (2012) reported homotypic dengue (DENV-1/2/3) infection increases IL-10 than heterotypic infection. In Tamil Nadu, circulation of DENV-2 was reported till 2017 (Kasirajan et al., 2019), and it is possible that the homotypic DENV-2-infected patient may contribute to the increased IL-10 in DENV-2 DF, while the heterotypic DENV-4 (a naïve population) infection has reduced IL-10.

IL-10 expression varies with respect to dengue structural protein E (Tsai et al., 2014) and non-structural proteins, particularly NS1 (Adikari et al., 2016) and NS3 (Malavige et al., 2013). Mutation in DENV-2 E protein was reported from Indian strains (Kumar et al., 2010; Kasirajan et al., 2019). There is also a possibility that mutated DENV-2 E protein might increase IL-10 expression during DF. Taken together, we hypothesize that the IL-10 elevation in these samples indicates it plays a role in DENV replication, and elevation during DF can be attributed to serotype-specific structural/non-structural dengue proteins and/or homotypic infection, which warrant further research.

#### IFNγ Expression in DENV Serotypes

IFNγ belongs to type II IFNs, an effector in cell-mediated immunity, and plays a role in antiviral activity [see review (Schroder et al., 2004; Kang et al., 2018)]. Some reports favor that IFNγ protects from dengue infection (Gunther et al., 2011; Prestwood et al., 2012); while others say it contributes to dengue pathogenesis (Kurane et al., 1989). IFNγ has been shown to increase in severe dengue (DHF/DSS) infection (Chakravarti and Kumaria, 2006; Pal et al., 2014; Povoa et al., 2016; Sehrawat et al., 2018). Experimental evidence shows increase of IFNγ during DENV infection (Prestwood et al., 2012; Tian et al., 2019). In our observation, IFNγ was increased significantly in all serotypes (**Figure 5E**), in pDENV-1, 2, and 4, in sDENV-1 and 2 (**Figure S1**), and in DENV-1 DF, DENV-2 DF, DHF and DSS, DENV-3 DHF, and DENV-4 DHF and DSS compared to control (**Figure S2**). Priyadarshini and colleagues (2010) have shown increased level of IFNγ level in DF, which is similar to our study. Previously, we have shown gradual increase in IFNγ mRNA and protein within dengue severity of primary and secondary infection (Gowri Sankar and Alwin Prem Anand, 2021). Significant upregulation was observed in primary infection compared to secondary infection of DENV-1, DENV-2, and DENV-4 (**Figure 6F**). Similarly, several research reports show increased IFNγ in primary than secondary infection (Chakravarti and Kumaria, 2006; Mangione et al., 2014; Singla et al., 2016); a possible explanation for increase in primary infection can be attributed to production of IFNγ by several cell types (Diamond et al., 2000; St John and Rathore, 2019), and decrease in secondary infection can be attributed to difference in epitopes recognizing dengue viral peptides from different serotypes [as reported by Simmons and colleagues (2005)], blocking of STAT1 by viral protein (NS4B) (Muñoz-Jordán et al., 2003), GM-CSF expression, and/or Th1/T17 shift (discussed below). This shows that irrespective of serotypes, IFNγ plays a significant role in dengue pathology.

#### TNFα Expression in DENV Serotypes

TNFα is an inflammatory cytokine that plays a significant role in immunity (Smith et al., 1994; Idriss and Naismith, 2000). In dengue fatalities, histopathology shows the virus triggered the pro-inflammatory cytokines TNFα along with IFNγ by mononuclear cell infiltration in liver, lung, and kidney (Povoa et al., 2016), which is evidence for cytokine storm–induced pathogenesis in dengue. From our observation, TNFα was significant and specifically observed in DENV-2 alone (**Figures 5D**, **6G**, **S1B**, **S2B**), where it was significantly upregulated in sDENV-2 than sDENV-4 (**Figure 6G**) and control (**Figure S1B**) and in DF than control (**Figure S2B**). The increased levels of TNFα have been reported in secondary heterotypic infection than homotypic (Sierra et al., 2012; Yang et al., 2016), indicating our samples also consist of heterotypic DENV-2 infection. Increased levels of TNFα have been reported in DF than control (Feitosa et al., 2016; Tuyen et al., 2020), indicating as a predictive factor for dengue severity (DHF/DSS).

TNFα and IFNγ act synergistically *via* mediator interferon regulatory factor (IRF)-1 [see review (Boehm et al., 1997)]. Experimental observations point out that in DENV-2 infection, higher levels of circulating TNFα, IFNγ, IRF-1 are observed as an innate immune response (Chen et al., 2013; Carlin et al., 2017). Taken together, TNFα and IFNγ are specific cytokines in DENV-2 infection pertaining to adaptive immunity.

#### IL-6 Expression in DENV Serotypes

IL-6 can act as pro- and anti-inflammatory cytokine (see review (Scheller et al., 2011; Schaper and Rose-John, 2015)). In severe dengue infection, IL-6 together with other cytokines (TNFα, IL-2, IL-8, IFNγ), induces vascular endothelial permeability and coagulopathy [see review (Lei et al., 2001; Leong et al., 2007)]. Interestingly, our observation points out IL-6 was significantly upregulated in DENV-2 than control and DENV-3 and 4 (**Figure 5B**). Levy and colleagues (2010) reported IL-6 association with DENV-2, which is similar to our result.

Several reports show elevated levels of IL-6 in DHF/DSS (Huang et al., 2000; Rachman and Rinaldi, 2006; Restrepo et al., 2008; Priyadarshini et al., 2010; Butthep et al., 2012; Cruz Hernandez et al., 2016; Masood et al., 2018; Wang et al., 2019) and in both (Arias et al., 2014), and the discrepancy might be due to infection by different serotype. Within primary and secondary DENV serotypes, IL-6 was significant in pDENV-2 than pDENV-3 and 4 (**Figure 6C**). With respect to dengue severity in serotypes, DHF in DENV-2 is significant than DENV-1 and 3 (**Figure 7F**), and DSS in DENV-1 is significantly elevated than DENV-4 (**Figure 7H**). Cruz Hernandez and colleagues (2016) observed significantly higher IL-6 and TNFα expression in DENV-2 than DENV-1 in primary infection; our observation is similar, where IL-6 and TNFα were significantly elevated in DENV-2 (**Figure S1**). Here we have shown IL-6 expression pattern is specific to DENV-2.

#### IL-8 Expression in DENV Serotypes

IL-8 plays a major role in triggering endothelial permeability (Talavera et al., 2004; Leong et al., 2007) in DENV infection. Elevated IL-8 has been reported in DHF (Raghupathy et al., 1998; Pandey et al., 2015; Iani et al., 2016). Similarly, in our observation, IL-8 was differentially expressed in DENV-4 alone with significant increase in primary infection than secondary (**Figures 6D**, **S1C**) and control (**Figure S1C**). Majority of the samples are from DHF (**Table S1**); when disease severity within serotypes was compared, IL-8 was significantly upregulated in DHF of DENV-3 and DENV-4, than DENV-2 (**Figure 7G**) and control (**Figures S2C, D**). Even though the sample size is smaller in DENV-3, significant increase has been observed only in DHF, indicating IL-8 is correlated with disease severity.

#### GM-CSF Expression in DENV Serotypes

In dengue infection, GM-CSF is associated with hypotension (Bozza et al., 2008; Lee et al., 2013) and inflammasome activation [reviewed in (Wu et al., 2013a)]. Few reports were available for GM-CSF expression in dengue infection (Bozza et al., 2008; Becquart et al., 2010; Lee et al., 2013; Wu et al., 2013a; Wu et al., 2013b; Patro et al., 2019). High levels of GM-CSF are seen in DENV-2 infection (Becquart et al., 2010; Lee et al., 2013; Wang et al., 2019; Patro et al., 2019) and in DENV-4 (Friberg et al., 2018). Secondary heterotypic infection in severe dengue (DHF) shows increased levels of GM-CSF (Bozza et al., 2008; Friberg et al., 2018; Patro et al., 2019; Wang et al., 2019). Previously, we have reported significantly increased GM-CSF expression in DF, DHF, and DSS of secondary infection (Gowri Sankar and Alwin Prem Anand, 2021). In this study, we found significant upregulation in DENV-2 and 4 than control (**Figure 5F**), in secondary DENV-1, 2, and 4 than primary infection (**Figure 6H**, **Figure S1**), in DENV-2 DF and DSS than control (**Figure S2B**), and in DENV-4 DHF and DSS than control (**Figure S2D**). Interestingly, GM-CSF and IFNγ show counterexpression in all serotypes during primary and secondary infection (**Figure S1**). Reports show IFNγ inhibits GM-CSF (Ozawa et al., 1996), and experimental evidence in mice model shows primary DENV infection is IFNγ-dependent and STAT1-independent (Shresta et al., 2005), which might be the reason for increased IFNγ and low GM-CSF during primary infection. GM-CSF inhibits IFNγ *via* STAT1 (Kasper et al., 2007); this might be the reason for low IFNγ in secondary infection, which needs to be studied. This is the first report on highly significant expression of GM-CSF in secondary DENV-1, 2, and 4, where it could be considered as a biomarker for secondary infection.

#### IL-1β Expression in DENV Serotypes

IL-1β belongs to pro-inflammatory cytokine which is crucial to host-defense mechanism against infection (Dinarello, 2018; Mantovani et al., 2019). In dengue infection, IL-1β increased in severe dengue (Suharti et al., 2002; Bozza et al., 2008; Wang et al., 2019; Pan et al., 2019a; Pan et al., 2019b), which is associated with thrombocytopenia (Bozza et al., 2008), tissue injury and vascular leakage (Pan et al., 2019a; Pan et al., 2019b), and fibrinolysis (Suharti et al., 2002). Experimental evidence shows ADE, a common factor in secondary infection or prior exposure to DENV infection, induces IL-1β synthesis (Callaway et al., 2015a; Cheung et al., 2018). In our finding, IL-1β was significantly elevated in DENV-2 (**Figure 5A**), sDENV-2 than sDENV-3 (**Figure 6A**), and in DENV-2 DF than sDENV-1 and 4 DF (**Figure 7A**). Callaway and colleagues (2015b) have shown ADE induces IL-1β in human monocyte cultures, which is similar to our studies where IL-1β increased in secondary infection. Recently, experimental evidence has shown DENV-2 non-structural proteins NS1 (Quirino-Teixeira et al., 2020), NS2A, and NS2B (Shrivastava et al., 2020) trigger IL-1β secretion, indicating our observation of elevated level of DENV-2 in DF is probably due to non-structural proteins (NS1, N2A, and N2B); however, further research is needed.

Interestingly, IL-1β was significantly downregulated in DENV-3 than control, DENV-2, and DENV-4 (**Figure 6A**). Recent observations have shown reduction of IL-1β is associated with low-density lipoprotein (LDL) (Marin-Palma et al., 2019); our observation might be also due to association of LDL and should be verified in the future.

#### IL-2 Expression in DENV Serotypes

In DENV infection, IL-2 is elevated in DF (Kurane et al., 1991) and DHF (Kurane et al., 1991; Leong et al., 2007) with a significant relationship with liver enzymes (Senaratne et al., 2016). IL-2 was reported to be elevated in sDENV infection (Hatch et al., 2011; Friberg et al., 2018); similarly, we have observed upregulation of IL-2 in sDENV-3 compared to pDENV-3 and sDENV-4 (**Figure 6B**). Experimental evidence on DENV-3 infection in animal model *Callithrix penicillata (* Ferreira et al., 2014) shows DENV-3 favors two axes of biomarker network, and IL-2/IL-6/monocytes/viremia is one among them. Hatch and colleagues (2011) have shown stimulation with DENV-4 antigen significantly increases IL-2 production in DENV-3, indicating heterotypic infection increases IL-2; our observation is similar and can be attributed to heterotypic infection. Kurane and colleagues (1991) have shown elevated IL-2 in Thai children; similar results were observed in DF of DENV-1 than DENV-2 (**Figure 7B**) and control (**Figure S2**). IL-2 increase can be attributed to serotype-specific dengue protein interaction and/or heterotypic infection, which warrant further research.

#### IP-10 Expression in DENV Serotypes

IP-10 is a chemokine expressed in response to IFN-α, β, γ, which play a major role in effector T cell generation and trafficking (Dufour et al., 2002). Recent reports show IP-10 has significant expression pattern in dengue-infected patients (Becquart et al., 2010; Malavige et al., 2012; Rathakrishnan et al., 2012; Zhao et al., 2016; Huang et al., 2018). Previously, we have shown significant increase in secondary DHF and DSS (Gowri Sankar and Alwin Prem Anand, 2021). In case of serotypes, IP-10 was higher in DENV-2 (Becquart et al., 2010), and no other reports were available for other serotypes. Interestingly, our study is the first to report IP-10 in DENV-4 infection where it is significantly higher in secondary infection than primary and control (**Figures 6I**, **S1C**) and in DSS (**Figure S2D**), where it can be used as a prognostic marker during secondary infection.

#### IL-4 Expression in DENV Serotypes

IL-4 induces mannose receptor (MR) on monocytes and macrophages, and MR is required for internalization of DENV (Miller et al., 2008; Miller et al., 2019). IL-4 is said to trigger Th2-type response (Kuczera et al., 2018) and is least studied in dengue pathology (Guabiraba and Ryffel, 2014). Experimental evidence points out pretreatment of monocytes with IL-4 will increase the susceptibility for DENV infection (Miller et al., 2008; Miller et al., 2019), indicating an increase in IL-4 facilitates viral entry/replication in macrophages. Similarly, when compared between different serotypes, increased IL-4 was observed in DF of DENV-2 than DENV-1 (**Figure 7C**), which can be attributed to IL-4 facilitating viral entry/replication.

IL-4 expression was higher in DHF than DF (Butthep et al., 2012; Wang et al., 2019). Similarly, in dengue severity, DENV-2 shows significant decrease of IL-4 in DSS than DHF (**Figure 8B**) and control (**Figure S2A**). It is a well-established fact that IL-4 and IFNγ antagonize each other [see review (Boehm et al., 1997; Paludan, 1998; Sprokholt et al., 2018)]. *In vitro* experiments show moDC grown in the presence of IL-4 and GM-CSF upon DENV infection inhibits IFNα/β and is incapable of priming T cells towards Th1; co-cultured T-cells produce small amount of IFNγ (Rodriguez-Madoz et al., 2010), indicating antagonizing effects against IFN. In our observation, decreased level of IL-4 and increased level of IFNγ were observed in DENV-1 and 2 (**Figures S2A, B**); this can be due to antagonizing nature of IFNγ over IL-4.

#### Differential Cytokine Expression Profile in DENV Serotypes Regarding Dengue Severity

The dengue severity can be interlinked to ADE/original antigenic sin with viral serotypes and genotypes, the host genetic makeup, patient’s age and gender, and viral proteins (Rothman, 2011; Guzman et al., 2016). In DENV infection, T cell plays a complex role in dengue severity [reviewed in (Tian et al., 2019)], and the cytokine expression shows the characteristic features of Th1, Th2 (Kurane et al., 1991; Chaturvedi et al., 2000; Ho et al., 2004; Hatch et al., 2011), and Th17 phenotypes (Wu et al., 2013). The Th1 cells produce IL-2, IFNγ, TNFα, IP-10, and GM-CSF; Th2 cells produce IL-4, 5, 6, 9, 10, 13, and 25; and Th17 cells produce IL-17A, 17F, 21, and 22 (Mosmann and Coffman, 1989; Dufour et al., 2002; Zhu and Paul, 2008). Interestingly, IL-6 and IL-10 were originally proposed as cytokine produced by Th2 cells but later found to be produced by both Th1 and Th2 cells [reviewed in (Moore et al., 2001; Diehl and Rincón, 2002; Trinchieri, 2007; Dienz and Rincon, 2009)]. Here we have compiled the differential cytokine profile in DENV serotypes for keen understanding on severity (DHF/DSS).

In DENV-1, marked increase in Th1 cytokines, IL-2 and IFNγ, was observed in DF than control (**Figure S2A**). IL-2 promotes Th1 differentiation and enhances IFNγ expression (Liao et al., 2011); thus the increase in IFNγ is possibly interlinked with IL-2. The Th2 cytokine IL-4 is downregulated in DF (**Figure S2A**), which is probably due to antagonizing nature of IFNγ over IL-4 (Paludan, 1998), triggering a shift towards Th1. The mannose structures in DENV E protein trigger IL-1β, IL-6, and IP-10 *via* DC-SIGN, leading to disease severity (Chen et al., 2008; Wang et al., 2011; Sprokholt et al., 2017; Sprokholt et al., 2018). The observed increased expression of IL-1β, IL-6, and IP-10 in DSS and DHF (**Figure 8A**) can be attributed to DENV E triggered cytokines, since majority of our samples are from primary infection (**Table S1**). Taken together, in DF Th1 cytokines IL-2 promote IFNγ expression, but ADE is not progressed *via* increase of Th2 cytokine. Instead it is suppressed by Th1 cytokine IFNγ and promotes the expression of IL-1β, IL-6, and IP-10, leading to DHF/DSS. We hypothesize that DENV proteins might be sufficient to elicit dengue severity (DHF/DSS) *via* activating cytokine-mediated immune response, indicating serotype-specific differential cytokine expression profile.

In DENV-2, significant increase in Th1 cytokines (IFNγ, TNFα, and GM-CSF) and Th1/2 cytokines (IL-6, IL-10) was observed in DF than control (**Figure S2B**). DENV-2 patients have increased levels of IL-6, IFNγ (Restrepo et al., 2008; Patro et al., 2019), IL-10, TNFα, and GM-CSF (Patro et al., 2019) in DF than control (**Figure S2**), which is similar to our observation. Experimental evidence also points out DENV-2 elicits increased expression of IL-6, IL-10, GM-CSF, and TNFα (Núñez-Avellaneda et al., 2018). In monocytes, increased expression of IL-10 can be triggered by DENV-2 infection or attachment and by NS4B (Tsai et al., 2014) and NS1 (Adikari et al., 2016). The NS5 of DENV-2 induces IL-8 (Medin et al., 2005; Rawlinson et al., 2009); our observation of increased IL-8 in DF can be attributed to this phenomenon of NS5 induction of IL-8, thus indicating DENV infection and its non-structural protein are sufficient to induce IL-6, IL-10, GM-CSF, IFNγ, and TNFα in DF.

Interestingly, we have observed decrease Th2 cytokines IL-4 and IL-10 and increase of Th1 cytokine GM-CSF in DSS compared to DHF in DENV-2 (**Figures 8B**, **S2B**). Experimental evidence in dendritic cells and macrophages (Wilbers et al., 2018) and DENV-infected GM-macrophages (Wu et al., 2013) have shown GM-CSF negatively regulates IL-10, indicating increase in GM-CSF reduces IL-10 in DSS. IFNγ strongly antagonizes IL-4 expression (Boehm et al., 1997), which is similar in our observation where gradual IFNγ increase and IL-4 decrease were observed (**Figure S2B**). In dengue severity, Th1 cytokines GM-CSF and IFNγ antagonize Th2 cytokines IL-10 and IL-4, respectively, for the progression towards DHF/DSS. Serotype-specific IL-1β and TNFα were also observed in DENV-2 (Hatch et al., 2011; Valero et al., 2014), which is similar in our observation (**Figures 5A, D**). Taken together, the differential cytokine expression pattern is specific to DENV-2 and might be triggered by DENV-2 non-structural protein.

In DENV-4, the pro-inflammatory IL-1β was significantly downregulated in DF than control (**Figure S2D**). Very few reports (Marin-Palma et al., 2019) are available for reduced IL-1β in DF patients. IL-1β is synergistically regulated along TREM1 (triggering receptor expressed on myeloid cells1) (Wu et al., 2012; Fan et al., 2016), an innate immune response molecule. Tolfvenstam and colleagues (2011) have shown TREM1 signaling in early dengue infection *via* transcriptional profiling. Ruiz-Pacheo and colleagues (2014) have shown TREM1 is downregulated in DF than control. Taken together, IL-1β can possibly be synergistically regulated along with TREM1 in DF.

Our observations show upregulation of Th1/pro-inflammatory cytokines IL-8, IFNγ, GM-CSF, and IP-10 in DHF/DSS than control (**Figure S2D**). Within dengue severity IL-1β, IL-10 (Th1/2), and IP-10 (Th1) have been upregulated in DHF/DSS than DF (**Figure S2D**), and IL-6 (Th1/Th2) and IL-17A (Th17) in DHF than DSS (**Figure 8C**). In DENV infection, IL-1β secretion requires NLRP3 and caspase1 activity (Wu et al., 2013; Callaway et al., 2015a; Callaway et al., 2015b; Malavige and Ogg, 2017; Pan et al., 2019b) and can be dependent (Callaway et al., 2015a) or independent of antibody signaling (Callaway et al., 2015b). Interestingly, recent reports show DENV-2 non-structural proteins M (Pan et al., 2019b), NS2A, and NS2B (Shrivastava et al., 2020) induce IL-1β production *via* NLRP3. Similarly, NS5 induces IL-8 expression (Medin et al., 2005). Since DENV-2 and DENV-4 are from the same ancestor, it is possible that the M, NS2A/B from DENV-4 can increase IL-1β, and NS5 can increase IL-8 in DHF/DSS, when majority of the samples are from primary infection (**Table S1**).

Another interesting fact in our observation is the involvement of proinflammatory IL-17A in DENV-4, which is not observed in other serotypes (**Figure 8C**). Elevated expression of IL-17A has been observed in DHF than DF (**Figure 8**); similar reports have been reported (Becquart et al., 2010; Fernando et al., 2016; Kuczera et al., 2016) that IL-17 has been associated with severe dengue. Acosta-Rodriguez and colleagues (2007) have reported activated monocytes and dendritic cells produce increased IL-1β and IL-6 that are essential for Th17 differentiation. Yang and colleagues (2011) have shown STAT3 (transcription factor) from IL-6 promotes IL-17A. Taken together, our observation shows similar pattern of increased IL-1β and IL-6 in DENV-4 patients (**Figure 8C**) can substantiate the raise of IL-17A in DHF cases.

However, IL-1β was reduced in DSS in comparison to DHF (**Figure 8C**). In human dendritic cells, the STAT3 activation *via* DENV infection was early in comparison to STAT5, and IFNγ enhances STAT3 (Ho et al., 2005), where the interaction of NS1 with STAT3 induces IL-6 (Chua et al., 2005) and can be related to the upregulation of IL-6 in DHF (**Figure 8C**). In humans Th subsets, Th1 cells mainly produce GM-CSF and not Th17 cells. IL-6 and IL-1β modulate GM-CSF-producing T cells from naïve T cells *via* STAT5 (Noster et al., 2014), and interestingly IL-6 was not influenced by STAT3/5, but STAT5 in itself can directly inhibit IL-17A production (Yang et al., 2011). Surprisingly, experimental evidence shows STAT5 was not suppressed in DENV1-4 serotypes (Zimmerman et al., 2019) and increased at later stages (Ho et al., 2005). Based on this, we hypothesize that the increased GM-CSF and decreased IL-17A were due to STAT5, thereby increased levels of GM-CSF suppress IL-17A in DSS.

Rajesh and colleagues (2019) have reported high cases of hepatomegaly in DENV-4 during the 2017 dengue epidemic in Tamil Nadu, which is similar to our observation (**Tables 1**, **S7**). The γδ T cells of the liver are the major contributor of IL-17 during DENV infection (Guabiraba and Ryffel, 2014). IL-17A was detectable in serum of severe DENV patients with hepatic damage (Fernando et al., 2016). Taken together, hepatomegaly might be associated with IL-17A in our DENV-4 patients.

DENV proteins play a major role in severe dengue [reviewed in (Ngono and Shresta, 2018)]. The virulence of DENV infection can be attributed to variation in dengue proteins. In DENV-2 genotype, T164S mutation in NS1 protein ascertains the fact that mutation in dengue protein can enhance clinical severity (Rodriguez-Roche et al., 2005), as well as increased Th1 response—IL-6, IFNγ, and TNFα—in *in vivo* and *in vitro* experimental model (Sierra et al., 2012; Chan et al., 2019). Similarly, DENV-4 genotype II (1976, DENV-4 1036 - KX812530) produces more Th1-related cytokine response than Th2 (Hamlin et al., 2017), while another DENV-4 genotype II (2013, LRV13/422 - KU513441) induces mixed Th1/Th17 immune response (Kuczera et al., 2016). Thus, we conclude the change in DENV genotype due to mutation in proteins alone can be a contributing factor to differential cytokine expression profile in different DENV serotype.

## Conclusion

In Tamil Nadu, all four serotypes of DENV are in existence of circulation from 1956 to 2017. Our phylogenetic analysis conclude (1) co-circulation of Asian and American/African of DENV-1 genotype in Madurai, Tamil Nadu, (2) establishment of Cosmopolitan genotype of DENV-2 in Tamil Nadu, (3) DENV-3 isolates formed genetically distinct genotype III and were closer to other Asian strains than Indian strains and, (4) DENV-4 as a dominant serotype during an outbreak in Tamil Nadu, since 1968 (49 years) with a shift in Clade C to D due to genetic distinction, which is first reported from this study.

We hypothesize that DHF/DSS in primary DENV infection in children might be serotype-specific ADE or due to prior JEV vaccination and in adults might be due to prior exposure to JEV. In DENV-4 there was increased DHF/DSS cases. It is possible viral proteins alone can trigger DHF/DSS; however, further studies are needed to confirm these hypotheses. Our analysis also revealed that there was no individual serotype-specific cytokine expression; however, serotype-specific cytokine profile was observed, i.e., in DENV-1, IL-1β, IL-6, and IP-10; in DENV-2, IL-4, IL-8, IL-10, IFNγ, and GM-CSF; and in DENV-4, IL-1β, IL-8, IL-10, IL-17A, IFNγ, GM-CSF, and IP-10. In dengue outbreaks, it is essential to identify circulating viral genotype and their fitness by mutational analysis to correlate with disease severity and immune status, as this correlation will be helpful in therapeutics applications.

## Data Availability Statement

The datasets presented in this study can be found in online repositories. The names of the repository/repositories and accession number(s) can be found in the article/**Supplementary Material**.

## Ethics Statement

The studies involving human participants were reviewed and approved by ICMR-CRME, Madurai, India. Written informed consent to participate in this study was provided by the participants’ legal guardian/next of kin.

## Author Contributions

Conceptualization, design, and methodology: SGS. Formal analysis and investigation: SGS, AAPA, and TM. Writing—original draft preparation: SGS and AAPA. Writing—review and editing: SGS, AAPA, and TM. Funding acquisition: SGS. All authors contributed to the article and approved the submitted version.

## Funding

The work was funded by DST-SERB Young Scientist project (YSS/2015/001847) and DHR-HRD Young Scientist project (DHR/HRD/YS-14-2015-16) to SGS.

## Author Disclaimer

All the authors state that the views expressed in this article are their own and not an official position of the institution or funder.

## Conflict of Interest

The authors declare that the research was conducted in the absence of any commercial or financial relationships that could be construed as a potential conflict of interest.

## Publisher’s Note

All claims expressed in this article are solely those of the authors and do not necessarily represent those of their affiliated organizations, or those of the publisher, the editors and the reviewers. Any product that may be evaluated in this article, or claim that may be made by its manufacturer, is not guaranteed or endorsed by the publisher.
