# Supplementary material for: Emergence of Dengue 4 as Dominant Serotype During 2017 Outbreak in South India and Associated Cytokine Expression Profile
Source: Front Cell Infect Microbiol. 2021 Aug 10;11:681937. doi: 10.3389/fcimb.2021.681937 (PMC8382982; doi:10.3389/fcimb.2021.681937)
Supplement: Supplementary file 1 [file DataSheet_1.docx]

**Emergence of Dengue 4 as dominant serotype during 2017 outbreak in South India and associated cytokine expression profile**

S. Gowri Sankar ^1^*, T. Mowna Sundari ^2,3^, A. Alwin Prem Anand ^4^*

^1^ICMR-Vector Control Research Center - Field Station, Madurai - 625002, Tamil Nadu, India.

^2^DBT - BIF Centre (Under DBT BTISNet Scheme), Lady Doak College, Madurai - 625002, Tamil Nadu, India.

^3^Department of Biotechnology, Lady Doak College, Madurai - 625002, Tamil Nadu, India.

^4^Institute of Clinical Anatomy and Cell Analysis, University of Tuebingen, Oesterbergstrasse 3, Tuebingen 72074, Germany.

*Corresponding authors

S. Gowri Sankar

ICMR-Vector Control Research Center - Field Station

No. 4, Sarojini Street

Chinna Chokkikulam

Madurai - 625002

Tamil Nadu, India

Ph. No.: + 91 9894158681

E-mail: sankar.immuno@gmail.com

ORCID ID: 0000-0001-8533-0815

A. Alwin Prem Anand

Institute of Clinical Anatomy and Cell Analysis

University of Tuebingen

Oesterbergstrasse 3

Tuebingen 72074

Germany

Ph. No.: + 49 17637048378

E-mail: alwinprem@gmail.com

ORCID ID: 0000-0002-5602-9422

Supplementary figures:

Fig.S1: Differential cytokine expression in primary and secondary infection in each serotypes.

The graph represents the differential cytokine expression profile in each serotype in primary and secondary infection with respect to control. The cytokines IL-10, IFNγ and GM-CSF shows significant variation in DENV-1 (A), IL-6, IL-10, IFNγ, TNFα, GM-CSF and IP-10 shows significant variation in DENV-2 (B) and, IL-8, IL-10, IFNγ, GM-CSF and IP-10 shows significant variation in DENV-4 (C). The significance was shown as asterix (*) - *p<*0.05, ** - *p<*0.01, *** - *p<*0.001, **** - *p<*0.0001. The error bars represent SEM. Control (N=20), Primary infection (N=49), Secondary infection (N=27).

Fig.S2: Differential cytokine expression in dengue severity in each serotypes with respect to control.

The graphical representations show the differential cytokine expression profile in each serotype within dengue severity (DF, DHF & DSS) in comparison to control. The cytokine variations was observed in IL-1β, IL-2, IL-4, IL-6, IFNγ and IP-10 for DENV-1 (A), in DENV-2, IL-4, IL-6, IL-10, IFNγ, TNFα and GM-CSF shows variation (B), in DENV-3 Il-8 and IFNγ alone shows differential expression pattern (C) and in DENV-4, IL-1β, IL-8, IL-10, IFNγ, GM-CSF and IP-10 shows variation in expression pattern (D). Statistical analysis was performed using Welch’s two-tailed *t* test and significance was marked as asterix (*) - *p<*0.05, ** - *p<*0.01, *** - *p<*0.001, **** - *p<*0.0001. The error bars represent SEM. Control (N=20), DENV-1 DF (N=4), DENV-2 DF (N=14), DENV-4 DF (N=3), DENV-1 DHF (N=5), DENV-2 DHF (N=5), DENV-3 DHF (N=4), DENV-4 DHF (N=21), DENV-1 DSS (N=5), DENV-2 DSS (N=3), DENV-3 DSS (N=3), DENV-4 DSS (N=9).

Supplementary Tables:

Table S1: Stratified data of serotype with respect to dengue severity and, primary and secondary infection

| **Serotype** | **Primary infection (N=49)** | | | **Secondary infection (N=27)** | | | **Grand total** |
| --- | --- | --- | --- | --- | --- | --- | --- |
|  | **DF (N=15)** | **DHF (N=24)** | **DSS (N=10)** | **DF (N=6)** | **DHF (N=11)** | **DSS (N=10)** |  |
| Age [Min. – Max. (Median)] | 7 – 67 (32) | 5 – 67 (44) | 7 – 67 (18.5) | 6 – 67 (29.5) | 6 – 23 (12) | 6 – 56 (11.5) | -- |
| Gender (Male:Female) | 8:7 | 13:11 | 4:6 | 2:4 | 4:7 | 4:6 | 76 |
| **Stratified serotype with age and gender** | | | | | | | |
| **DENV-1** | | | | | | | |
| Children (5-18yrs) – Female | 3 | 0 | 0 | 0 | 1 | 0 |  |
| Children (5-18yrs) – Male | 0 | 0 | 1 | 0 | 1 | 2 |  |
| Adult (19-67yrs) – Female | 0 | 2 | 0 | 0 | 0 | 0 |  |
| Adult (19-67yrs) – Male | 1 | 1 | 2 | 0 | 0 | 0 |  |
| Total (%)^†^ | 4 (28.57) | 3 (21.43) | 3 (21.43) | 0 | 2 (14.29) | 2 (14.29) | 14 |
| **DENV-2** | | | | | | | |
| Children (5-18yrs) – Female | 1 | 0 | 0 | 1 | 1 | 1 |  |
| Children (5-18yrs) – Male | 1 | 0 | 0 | 1 | 1 | 0 |  |
| Adult (19-67yrs) – Female | 3 | 1 | 1 | 1 | 0 | 0 |  |
| Adult (19-67yrs) – Male | 4 | 2 | 0 | 2 | 0 | 1 |  |
| Total (%)^†^ | 9 (40.91) | 3 (13.64) | 1 (4.55) | 5 (22.73) | 2 (9.09) | 2 (9.09) | 22 |
| **DENV-3** | | | | | | | |
| Children (5-18yrs) – Female | 0 | 0 | 0 | 0 | 0 | 0 |  |
| Children (5-18yrs) – Male | 0 | 0 | 1 | 0 | 2 | 0 |  |
| Adult (19-67yrs) – Female | 0 | 0 | 1 | 0 | 0 | 0 |  |
| Adult (19-67yrs) – Male | 0 | 2 | 0 | 0 | 0 | 1 |  |
| Total (%)^†^ | 0 | 2 (28.57) | 2 (28.57) | 0 | 2 (28.57) | 1 (14.29) | 7 |
| **DENV-4** | | | | | | | |
| Children (5-18yrs) – Female | 0 | 5 | 2 | 0 | 2 | 3 |  |
| Children (5-18yrs) – Male | 0 | 1 | 1 | 1 | 2 | 1 |  |
| Adult (19-67yrs) – Female | 1 | 5 | 0 | 0 | 0 | 0 |  |
| Adult (19-67yrs) – Male | 1 | 5 | 1 | 0 | 1 | 1 |  |
| Total (%)^†^ | 2 (6.06) | 16 (48.48) | 4 (12.12) | 1 (3.03) | 5 (15.15) | 5 (15.15) | 33 |
| Total – Children^$^ (%) | 5 (6.58) | 6 (7.89) | 5 (6.58) | 3 (3.95) | 10 (13.16) | 7 (9.21) |  |
| Total – Adult^#^ (%) | 10 (13.16) | 18 (23.68) | 5 (6.58) | 3 (3.95) | 1 (1.32) | 3 (3.95) |  |
| Grand total* (%) | 15 (19.74) | 24 (31.58) | 10 (13.16) | 6 (7.89) | 11 (14.47) | 10 (13.16) | 76 |

^†^The percentage was calculated with the sum total value of respective samples within each serotype DF/DHF/DSS with their respective total number of samples (Ex. For DENV-1, sum of pDF/total no. of DENV-1 samples * 100)

^$^Total children was obtained by adding values of children samples from all DENV serotypes and, percentage was calculated from total sample size i.e., 76.

^#^Total adult was obtained by adding the values of adult samples from all DENV serotypes and, percentage was calculated from total sample size i.e., 76.

*Grand total was obtained by adding values of DF, DHF and DSS from DENV-1, DENV-2, DENV-3 and DENV-4 and, percentage was calculated from the total sample size i.e., 76.

Abbr.: DENV – dengue virus, DF – dengue fever, DHF – dengue haemorrhagic fever, DSS – dengue shock syndrome

Table S2: List of sequences obtained from this study with their accession numbers.

| S. No. | Sample ID | Accession No. | Serotype | Clinical parameter | |
| --- | --- | --- | --- | --- | --- |
|  |  |  |  | Stage | Phase |
|  | MDU122 | LR595969 | Dengue 1 | Primary | DHF |
|  | MDU45 | LR595964 | Dengue 1 | Secondary | DHF |
|  | MDU58 | LR595965 | Dengue 1 | Primary | DHF |
|  | MDU75 | LR595966 | Dengue 1 | Primary | DSS |
|  | MDU123 | LR595970 | Dengue 1 | Secondary | DHF |
|  | MDU78 | LR595967 | Dengue 1 | Primary | DSS |
|  | MDU99 | LR595968 | Dengue 1 | Secondary | DHF |
|  | MDU140 | LR595983 | Dengue 2 | Primary | DHF |
|  | MDU77 | LR595977 | Dengue 2 | Secondary | DHF |
|  | MDU23 | LR595971 | Dengue 2 | Primary | DF |
|  | MDU44 | LR595973 | Dengue 2 | Primary | DHF |
|  | MDU71 | LR595974 | Dengue 2 | Primary | DF |
|  | MDU85 | LR595978 | Dengue 2 | Secondary | DF |
|  | MDU72 | LR595975 | Dengue 2 | Secondary | DHF |
|  | MDU94 | LR595980 | Dengue 2 | Primary | DHF |
|  | MDU33 | LR595972 | Dengue 2 | Primary | DF |
|  | MDU93 | LR595979 | Dengue 2 | Primary | DF |
|  | MDU73 | LR595976 | Dengue 2 | Secondary | DF |
|  | MDU102 | LR595982 | Dengue 2 | Primary | DF |
|  | MDU97 | LR595981 | Dengue 2 | Secondary | DF |
|  | MDU11 | LR595984 | Dengue 3 | Secondary | DSS |
|  | MDU100 | LR595985 | Dengue 3 | Primary | DHF |
|  | MDU104 | LR596001 | Dengue 4 | Primary | DSS |
|  | MDU105 | LR596002 | Dengue 4 | Secondary | DF |
|  | MDU92 | LR596000 | Dengue 4 | Primary | DHF |
|  | MDU69 | LR595997 | Dengue 4 | Primary | DSS |
|  | MDU67 | LR595995 | Dengue 4 | Secondary | DHF |
|  | MDU48 | LR595992 | Dengue 4 | Primary | DF |
|  | MDU40 | LR595991 | Dengue 4 | Secondary | DHF |
|  | MDU37 | LR595990 | Dengue 4 | Primary | DSS |
|  | MDU22 | LR595988 | Dengue 4 | Primary | DHF |
|  | MDU112 | LR596003 | Dengue 4 | Primary | DSS |
|  | MDU119 | LR596004 | Dengue 4 | Primary | DF |
|  | MDU130 | LR596005 | Dengue 4 | Primary | DHF |
|  | MDU90 | LR595999 | Dengue 4 | Secondary | DSS |
|  | MDU68 | LR595996 | Dengue 4 | Primary | DHF |
|  | MDU79 | LR595998 | Dengue 4 | Secondary | DSS |
|  | MDU12 | LR595986 | Dengue 4 | Primary | DHF |
|  | MDU35 | LR595989 | Dengue 4 | Primary | DHF |
|  | MDU16 | LR595987 | Dengue 4 | Secondary | DSS |
|  | MDU61 | LR595994 | Dengue 4 | Secondary | DSS |
|  | MDU49 | LR595993 | Dengue 4 | Secondary | DSS |

Table S3: List of sequences retrieved from GenBank and used for phylogenetic analysis

| Serotype | Genotype | Accession Number | Country | Year |
| --- | --- | --- | --- | --- |
| Dengue 1 | GI-Asian | AY584593 | India | 1997 |
|  |  | AY584594 | India | 1998 |
|  |  | EU848585 | USA | 1944 |
|  |  | AB074760 | Japan | 1943 |
|  |  | M87512 | Singapore | 1990 |
|  |  | AY726555 | Myanmar | 1998 |
|  |  | AY726552 | Myanmar | 2002 |
|  |  | AF350498 | China | 1980 |
|  |  | GQ199833 | Vietnam | 2004 |
|  |  | GU131923 | Cambodia | 2005 |
|  |  | FJ410249 | Vietnam | 2008 |
|  |  | AF309641 | Cambodia | 1998 |
|  |  | AY732479 | Thailand | 2001 |
|  |  | DQ193572 | China | 2004 |
|  |  | KJ755855 | India | 2013 |
|  |  | EU280167 | China | 2006 |
|  |  | HQ891313 | Srilanka | 2003 |
|  |  | JN0542555 | Srilanka | 2010 |
|  |  | KT445959 | Srilanka | 2012 |
|  |  | KT827365 | China | 2006 |
|  |  | KY586439 | Thailand | 2006 |
|  | GII- Thailand | AF180818 | Thailand | 1964 |
|  |  | JG922547 | Thailand | 1960 |
|  | GIII- Sylvatic | EF457905 | Malaysia | 1972 |
|  | GIV- South Pacific | FJ196842 | China | 2003 |
|  |  | KT827364 | USA | 2001 |
|  |  | EF025110 | China | 2002 |
|  |  | AB189121 | Indonesia | 1998 |
|  |  | AB204803 | Japan | 2004 |
|  |  | DQ285560 | Reunion | 2004 |
|  |  | AB195673 | Japan | 2004 |
|  |  | DQ285561 | Seychelles | 2004 |
|  |  | FJ196846 | China | 1995 |
|  |  | EU863650 | Chile | 2002 |
|  |  | JQ915077 | New Caledonia | 2002 |
|  |  | JQ915076 | French Polynesia | 2009 |
|  |  | JQ915080 | New Caledonia | 2010 |
|  | GV- American/African | KX458397 | China | 2014 |
|  |  | KX098540 | India | 2014 |
|  |  | KX380803 | Singapore | 2013 |
|  |  | JQ917404 | India | 2009 |
|  |  | KM403575 | Singapore | 2012 |
|  |  | JN903581 | India | 2009 |
|  |  | JN903579 | India | 2008 |
|  |  | KU948532 | India | 2015 |
|  |  | GQ357692 | Singapore | 2008 |
|  |  | JQ922545 | India | 1982 |
|  |  | AY732476 | Thailand | 1980 |
|  |  | DQ285562 | Comoros | 1993 |
|  |  | KJ806950 | Singapore | 2013 |
|  |  | EU846230 | India | 2007 |
|  |  | KP406801 | Korea | 2004 |
|  |  | EU181199 | India | 2006 |
|  |  | EU181195 | India | 2006 |
|  |  | EU181194 | India | 2006 |
|  |  | EU626491 | India | 2004 |
|  |  | EU846233 | India | 2007 |
|  |  | DQ285559 | Reunion | 2004 |
|  |  | JN903578 | India | 2007 |
|  |  | FJ547086 | Puerto Rico | 1996 |
|  |  | AF226686 | French Guyana | 1989 |
|  |  | GQ868561 | Columbia | 1999 |
|  |  | FJ639735 | Venezuela | 1997 |
|  |  | EF222444 | India | 2005 |
|  |  | EF064774 | India | 2005 |
|  |  | EF064776 | India | 2005 |
|  |  | AY593214 | India | 1964 |
|  |  | AY593213 | India | 1963 |
|  |  | AY593210 | India | 1962 |
|  | GVI-Sylvatic | KR919820 | Brunei | 2014 |
| Dengue 2 | GI-Asian II | AF204178 | China | 1987 |
|  |  | AF204177 | China | 1989 |
|  | GII-Asian I | AF022437 | Japan | 1999 |
|  |  | AF469176 | China | 1998 |
|  |  | AF100464 | Thailand | 1996 |
|  | GIII- Asian/American | AF489932 | Brazil | 1998 |
|  |  | GQ398314 | Puerto Rico | 1994 |
|  | GIV-Cosmopolitan | KU948525 | India | 2015 |
|  |  | GQ340960 | India | 2008 |
|  |  | GQ340961 | India | 2008 |
|  |  | KJ438869 | India | 2012 |
|  |  | GU968539 | India | 2009 |
|  |  | JN935383 | India | 2010 |
|  |  | KX577715 | China | 2015 |
|  |  | KT180235 | India | 2013 |
|  |  | DQ448237 | India | 2006 |
|  |  | AF047401 | India | 1996 |
|  |  | AY593218 | India | 1993 |
|  |  | GQ252677 | Srilanka | 2004 |
|  |  | KF360005 | Pakistan | 2010 |
|  |  | KJ010186 | Pakistan | 2013 |
|  |  | JQ955623 | India | 2009 |
|  |  | KF041236 | Pakistan | 2009 |
|  |  | AF359579 | China | 1999 |
|  |  | FJ882602 | Srilanka | 1996 |
|  |  | JN935392 | India | 2010 |
|  |  | JX475906 | India | 2009 |
|  |  | JN935393 | India | 2010 |
|  |  | KU948527 | India | 2015 |
|  |  | KT180259 | India | 2014 |
|  |  | KT180260 | India | 2014 |
|  |  | JN935391 | India | 2010 |
|  |  | AY706094 | India | 2003 |
|  |  | KM274897 | India | 2013 |
|  |  | KY404147 | India | 2014 |
|  |  | KY404138 | India | 2014 |
|  |  | KY550236 | India | 2016 |
|  |  | KY550240 | India | 2016 |
|  |  | GQ398263 | Indonesia | 1975 |
|  |  | JX470186 | China | 2010 |
|  |  | AY776328 | Taiwan | 2004 |
|  |  | EU179858 | Brunei | 2005 |
|  |  | EU081180 | Singapore | 2005 |
|  |  | AY037116 | Australia | 1993 |
|  |  | AY858035 | Indonesia | 2004 |
|  | GV- American | AY593227 | India | 1974 |
|  |  | AY593228 | India | 1956 |
|  |  | AY593226 | India | 1971 |
| Dengue 3 | GI | AY744685 | French Polynesia | 1994 |
|  |  | JQ920486 | New Caledonia | 1996 |
|  |  | AY744863 | French Polynesia | 1992 |
|  |  | AY744677 | French Polynesia | 1989 |
|  |  | FJ898456 | Samoa | 1995 |
|  |  | DQ401690 | Indonesia | 1982 |
|  |  | AB214882 | East Timor | 2005 |
|  |  | DQ675519 | Taiwan | 1995 |
|  |  | AY496879 | Philippines | 1997 |
|  | GII | KU509280 | Thailand | 2011 |
|  |  | KU509284 | Thailand | 2008 |
|  |  | AY766104 | Singapore | 1995 |
|  |  | GQ868629 | Cambodia | 2005 |
|  |  | KF955461 | Cambodia | 1999 |
|  |  | DQ675522 | Taiwan | 1998 |
|  |  | KF955462 | Cambodia | 2000 |
|  |  | FJ639719 | Cambodia | 2000 |
|  |  | AY912458 | Thailand | 1998 |
|  |  | GQ868593 | Thailand | 1973 |
|  |  | KF955477 | India | 1984 |
|  |  | JN406514 | Australia | 1998 |
|  | GIII | GQ252674 | Srilanka | 1997 |
|  |  | JQ922555 | Vellore, India | 1966 |
|  |  | FJ882571 | Srilanka | 1989 |
|  |  | KF955465 | Puerto Rico | 2000 |
|  |  | KF955468 | Puerto Rico | 2001 |
|  |  | GU370053 | Singapore | 2007 |
|  |  | FJ882574 | Srilanka | 1985 |
|  |  | GQ199889 | Srilanka | 1983 |
|  |  | FJ882572 | Srilanka | 1989 |
|  |  | EU081198 | Singapore | 2005 |
|  |  | EU081181 | Singapore | 2004 |
|  |  | HQ166304 | Nicaragua | 1009 |
|  |  | EU8524292 | Venezuela | 2005 |
|  |  | KT726344 | Cuba | 2001 |
|  |  | KT726348 | Cuba | 2002 |
|  |  | KU216209 | India | 2013 |
|  |  | EF546774 | India | 2006 |
|  |  | EF846234 | India | 2007 |
|  |  | KJ451722 | India | 2012 |
|  |  | KF954949 | China | 2013 |
|  |  | KF954947 | China | 2013 |
|  |  | KX380842 | Singapore | 2012 |
|  |  | KX380841 | Singapore | 2012 |
|  |  | JN940916 | India | 2010 |
|  |  | FJ644564 | India | 2007 |
|  |  | KF041259 | Pakistan | 2006 |
|  |  | GU363549 | China | 2009 |
|  |  | JN940914 | India | 2010 |
|  |  | AY770511 | India | 2003 |
|  |  | JX070120 | India | 2010 |
|  |  | JX070123 | India | 2010 |
|  |  | AY662691 | Singapore | 2005 |
|  |  | AY099336 | Srilanka | 2000 |
|  |  | DQ675533 | Taiwan | 1999 |
|  |  | FJ882573 | Srilanka | 1993 |
|  |  | JX669494 | Brazil | 2005 |
|  |  | JX669508 | Brazil | 2006 |
|  | GV | EF629370 | Brazil | 2002 |
|  |  | AF317645 | China | 2000 |
|  |  | M93130 | Philippines | 1956 |
| DENV 4 | GI | AF289029 | China | 1990 |
|  |  | AY550909 | Srilanka | 1978 |
|  |  | AY618990 | Thailand | 1991 |
|  |  | AY618991 | Thailand | 1997 |
|  |  | AY618992 | Thailand | 2001 |
|  |  | AY947539 | Philippines | 1956 |
|  |  | EU652498 | India | 2007 |
|  |  | EU652500 | India | 2007 |
|  |  | FJ196850 | China | 1990 |
|  |  | GQ868594 | Philippines | 1956 |
|  |  | JN638572 | Cambodia | 2008 |
|  |  | JN882277 | India | 2010 |
|  |  | JQ513345 | Brazil | 2011 |
|  |  | JQ639486 | India | 2010 |
|  |  | JQ922559 | India | 1979 |
|  |  | JQ922560 | India | 2009 |
|  |  | KF041260 | Pakistan | 2009 |
|  |  | KF543373 | India | 2011 |
|  |  | KF955510 | Cambodia | 2002 |
|  |  | KX059025 | Srilanka | 2013 |
|  |  | KX059027 | Srilanka | 2013 |
|  |  | KX059037 | Srilanka | 2013 |
|  |  | KX845005 | India | 2015 |
|  |  | KY315119 | India | 2016 |
|  |  | MF489721 | India | 2015 |
|  |  | MG053162 | Pune, India | 2016 |
|  |  | MG053163 | Pune, India | 2016 |
|  |  | MG053164 | Pune, India | 2016 |
|  |  | MG053165 | Pune, India | 2016 |
|  |  | MG053166 | Pune, India | 2016 |
|  |  | MG053167 | Pune, India | 2016 |
|  |  | MG053168 | Pune, India | 2016 |
|  |  | MG053169 | Pune, India | 2016 |
|  |  | MG053170 | Pune, India | 2016 |
|  |  | MG053171 | Pune, India | 2016 |
|  |  | MG053172 | Pune, India | 2016 |
|  |  | MG053173 | Pune, India | 2016 |
|  |  | MG272272 | Pune, India | 2016 |
|  |  | MG491383 | Karnataka, India | 2015 |
|  |  | MG491384 | Andra Pradesh, India | 2015 |
|  |  | MG491385 | Karnataka, India | 2015 |
|  |  | MG491386 | Andra Pradesh, India | 2015 |
|  |  | MG491387 | Karnataka, India | 2015 |
|  |  | MG491388 | Karnataka, India | 2015 |
|  |  | MG491389 | Andra Pradesh, India | 2015 |
|  |  | MH431917 | Pondicherry & Tamilnadu, India | 2017 |
|  |  | MH431918 | Pondicherry & Tamilnadu, India | 2017 |
|  |  | MH431919 | Pondicherry & Tamilnadu, India | 2017 |
|  |  | MH431920 | Pondicherry & Tamilnadu, India | 2017 |
|  |  | MH431921 | Pondicherry & Tamilnadu, India | 2017 |
|  |  | MH431922 | Pondicherry & Tamilnadu, India | 2017 |
|  |  | MH431923 | Pondicherry & Tamilnadu, India | 2017 |
|  |  | MH431924 | Pondicherry & Tamilnadu, India | 2017 |
|  |  | MH431925 | Pondicherry & Tamilnadu, India | 2017 |
|  |  | MH891769 | Krishnagiri, Tamil Nadu, India | 2017 |
|  |  | MK603192 | Vellore, India | 2017 |
|  |  | MK829120 | Central India | 2018 |
|  |  | MN365201 | Mumbai, India | 2018 |
|  |  | MN365214 | Mumbai, India | 2018 |
|  |  | MN365219 | Mumbai, India | 2018 |
|  |  | MN365229 | Mumbai, India | 2018 |
|  |  | MN365230 | Mumbai, India | 2018 |
|  | GII | KU513441 | Brazil | 2013 |
|  |  | JN983813 | Brazil | 2010 |
|  |  | FJ639738 | Venezuela | 1998 |
|  |  | FJ882590 | Venezuela | 2007 |
|  |  | GU289913 | Columbia | 1982 |
|  |  | AH012032 | Puerto Rico | 1981 |
|  |  | AH011997 | Puerto Rico | 1987 |
|  |  | AF326573 | Dominica | 1981 |
|  |  | GU318316 | USA | 1985 |
|  |  | AH011980 | Puerto Rico | 1994 |
|  |  | KF907503 | Senegal | 1953 |
|  | GIII | AY618988 | Thailand | 1997 |
|  |  | AY618989 | Thailand | 1997 |
|  | GIV | JF262780 | Malaysia | 1973 |
|  | GV | JF262783 | Vellore, India | 1961 |
|  |  | JQ922558 | Vellore, India | 1962 |
|  | GVI | KC762698 | Indonesia | 2008 |
|  |  | JQ822247 | China | 2010 |
|  |  | LC069810 | Japan | 2016 |
|  |  | JX024757 | Singapore | 2010 |
|  |  | AY618993 | Thailand | 2000 |
|  |  | AY776330 | Taiwan | 2000 |
|  |  | KC762675 | Indonesia | 2007 |

Table S4: Cytokine expression between control and serotypes

| Cytokine | Control (N=20) | DENV1 (N=14) | DENV2 (N=22) | DENV3  (N=7) | DENV4 (N=33) | Control vs. DENV1 | Control vs. DENV2 | Control vs. DENV3 | Control vs. DENV4 | DENV1 vs. DENV2 | DENV1 vs. DENV3 | DENV1 vs. DENV4 | DENV2 vs. DENV3 | DENV2 vs. DENV4 | DENV3 vs. DENV4 |
| --- | --- | --- | --- | --- | --- | --- | --- | --- | --- | --- | --- | --- | --- | --- | --- |
|  | Samples | | | | | *p* Value | | | | | | | | | |
| IL1β | 1.99±1.63 | 1.67±0.91 | 2.02±1.17 | 1.03±0.66 | 1.93±1.18 | 0.48 | 0.94 | **0.04** | 0.89 | 0.32 | 0.09 | 0.43 | **0.01** | 0.77 | **0.01** |
| IL2 | 2.58±2.12 | 3.11±1.55 | 2.87±1.29 | 2.76±2.08 | 2.76±1.38 | 0.40 | 0.60 | 0.85 | 0.73 | 0.63 | 0.70 | 0.47 | 0.89 | 0.77 | 0.99 |
| IL4 | 4.55±2.82 | 3.89±1.81 | 4.05±1.53 | 4.9±2.08 | 4.19±1.6 | 0.41 | 0.48 | 0.74 | 0.60 | 0.78 | 0.30 | 0.59 | 0.35 | 0.75 | 0.42 |
| IL6 | 22.85±11.15 | 27.36±6.77 | 30.77±8.42 | 22.5±5.24 | 25.58±8.04 | 0.15 | **0.01** | 0.91 | 0.35 | 0.19 | 0.09 | 0.45 | **0.01** | **0.03** | 0.23 |
| IL8 | 58.78±20.15 | 64.07±16.15 | 61.54±24.7 | 70.94±18.28 | 68.09±20.79 | 0.40 | 0.69 | 0.17 | 0.11 | 0.71 | 0.42 | 0.48 | 0.30 | 0.31 | 0.72 |
| IL10 | 25.25±16.01 | 42.01±16.78 | 35.37±12.5 | 31.61±9.7 | 33.22±11.96 | **0.01** | **0.03** | 0.23 | 0.06 | 0.22 | 0.09 | 0.09 | 0.42 | 0.53 | 0.71 |
| IL12 | 40.99±28.74 | 47.33±14.43 | 45.2±11.6 | 42.64±11.73 | 42.89±12.47 | 0.40 | 0.55 | 0.83 | 0.78 | 0.65 | 0.44 | 0.33 | 0.63 | 0.49 | 0.96 |
| IL17A | 3.08±2.91 | 3.79±2.28 | 3.48±2 | 4.13±1.56 | 3.35±1.94 | 0.43 | 0.61 | 0.25 | 0.71 | 0.67 | 0.70 | 0.53 | 0.39 | 0.82 | 0.28 |
| IFNγ | 15±5.25 | 33.01±14.72 | 32.05±11.93 | 31.43±11.83 | 28.88±11.86 | **0.001** | **<0.0001** | **0.01** | **<0.0001** | 0.84 | 0.79 | 0.36 | 0.91 | 0.34 | 0.62 |
| TNFα | 19.01±3.01 | 22.42±9.71 | 22.76±7.48 | 17.41±6.81 | 18.76±7.63 | 0.22 | **0.04** | 0.57 | 0.87 | 0.91 | 0.19 | 0.22 | 0.10 | 0.06 | 0.65 |
| GM-CSF | 2.46±1.79 | 3.7±2.17 | 4.58±2.19 | 2.99±1.92 | 4.24±1.97 | 0.09 | **0.001** | 0.08 | **0.002** | 0.24 | 0.42 | 0.43 | 0.93 | 0.56 | 0.70 |
| IP10 | 708.11±189.15 | 656.67±186.69 | 766.03±197.5 | 874.77±229.49 | 777.25±187.36 | 0.44 | 0.34 | 0.12 | 0.20 | 0.10 | 0.05 | 0.05 | 0.29 | 0.83 | 0.32 |

The cytokine expression was given in mean±SD. The statistical analysis was carried out using two-tailed t test and significant values are given in bold.

Abbr.: IL – interleukin, IFNγ – interferon gamma, TNFα – tumour necrosis factor alpha, GM-CSF – granulocyte macrophage colony stimulating factor, IP – interferon gamma-inducible protein.

Table S5: Cytokines expression among different serotypes in primary and secondary infection

| Cytokine | Control (N=20) | Primary infection | | | | Secondary infection | | | |
| --- | --- | --- | --- | --- | --- | --- | --- | --- | --- |
|  |  | DENV1 (N=10) | DENV2 (N=13) | DENV3 (N=4) | DENV4 (N=22) | DENV1 (N=4) | DENV2 (N=9) | DENV3 (N=3) | DENV4 (N=11) |
| IL1β | 1.99±1.63 | 1.42±0.82 | 1.76±1.18 | 1.16±0.72 | 2.00±1.17 | 2.30±0.92 | 2.39±1.11 | 0.87±0.67 | 1.77±1.24 |
| IL2 | 2.58±2.12 | 3.37±1.55 | 2.87±1.46 | 1.40±1.47 | 3.09±1.35 | 2.48±1.58 | 2.88±1.08 | 4.57±1.08 | 2.11±1.26 |
| IL4 | 4.55±2.82 | 3.94±2.08 | 4.04±1.83 | 6.00±2.07 | 4.09±1.62 | 3.75±1.10 | 4.07±1.07 | 3.43±0.92 | 4.38±1.61 |
| IL6 | 22.85±11.15 | 28.47±5.82 | 33.35±9.35 | 21.58±3.25 | 26.21±8.46 | 24.57±9.08 | 27.04±5.35 | 23.73±7.92 | 24.33±7.37 |
| IL8 | 58.78±20.15 | 65.51±17.55 | 61.32±28.35 | 73.83±17.76 | 73.78±22.47 | 60.48±13.48 | 61.86±19.92 | 67.10±22.15 | 56.72±10.44 |
| IL10 | 25.25±16.01 | 42.54±17.02 | 38.67±11.41 | 35.35±8.15 | 35.40±12.32 | 40.68±18.65 | 30.61±13.11 | 26.63±10.83 | 28.87±10.38 |
| IL12 | 40.99±28.74 | 46.12±12.98 | 49.23±12.08 | 49.68±7.53 | 46.36±12.45 | 50.35±19.50 | 39.38±8.36 | 33.27±9.84 | 35.95±9.63 |
| IL17A | 3.08±2.91 | 3.10±1.24 | 3.47±1.77 | 4.43±1.79 | 3.71±2.09 | 5.53±3.50 | 3.49±2.42 | 3.73±1.44 | 2.64±1.42 |
| IFNγ | 15.00±5.25 | 38.44±14.02 | 36.31±11.87 | 33.30±15.42 | 33.70±10.06 | 19.45±2.42 | 25.91±9.52 | 28.93±6.87 | 19.26±9.22 |
| TNFα | 19.01±3.01 | 21.13±11.07 | 21.52±8.54 | 13.83±6.43 | 19.43±8.72 | 25.65±4.65 | 24.57±5.59 | 22.20±4.11 | 17.41±4.88 |
| GM-CSF | 2.46±1.79 | 2.56±1.14 | 3.21±1.55 | 3.23±2.05 | 3.35±1.51 | 6.53±1.25 | 6.57±1.22 | 6.63±2.48 | 6.04±1.51 |
| IP-10 | 708.11±189.15 | 607.18±172.37 | 672.58±159.09 | 734.23±126.95 | 709.09±182.88 | 780.39±182.54 | 901.00±172.45 | 1062.17±204.06 | 913.57±106.93 |

The cytokine expression was given in mean±SD.

Abbr.: IL – interleukin, IFNγ – interferon gamma, TNFα – tumour necrosis factor alpha, GM-CSF – granulocyte macrophage colony stimulating factor, IP – interferon gamma-inducible protein.Table S5.1: Statistical comparison of cytokine level between control and different serotypes in primary and secondary infection

| Cytokine | Primary infection | | | | Secondary infection | | | |
| --- | --- | --- | --- | --- | --- | --- | --- | --- |
|  | Control vs. DENV1 | Control vs. DENV2 | Control vs. DENV3 | Control vs. DENV4 | Control vs. DENV1 | Control vs. DENV2 | Control vs. DENV3 | Control vs. DENV4 |
| IL1β | 0.22 | 0.66 | 0.14 | 0.97 | 0.61 | 0.45 | 0.07 | 0.69 |
| IL2 | 0.26 | 0.65 | 0.23 | 0.36 | 0.91 | 0.62 | >0.05 | 0.45 |
| IL4 | 0.51 | 0.53 | 0.28 | 0.52 | 0.35 | 0.51 | 0.21 | 0.83 |
| IL6 | 0.08 | **0.01** | 0.67 | 0.28 | 0.75 | 0.18 | 0.88 | 0.66 |
| IL8 | 0.36 | 0.78 | 0.19 | **0.03** | 0.84 | 0.71 | 0.59 | 0.71 |
| IL10 | **0.02** | **0.01** | 0.10 | **0.03** | 0.20 | 0.35 | 0.86 | 0.45 |
| IL12 | 0.51 | 0.27 | 0.26 | 0.45 | 0.45 | 0.82 | 0.39 | 0.48 |
| IL17A | 0.98 | 0.63 | 0.26 | 0.43 | 0.26 | 0.70 | 0.56 | 0.58 |
| IFNγ | **0.0004** | **1.98178E-05** | 0.10 | **9.71859E-09** | **0.02** | **0.009** | 0.06 | 0.18 |
| TNFα | 0.57 | 0.33 | 0.21 | 0.83 | 0.06 | **0.02** | 0.31 | 0.34 |
| GM-CSF | 0.85 | 0.21 | 0.53 | 0.09 | **0.002** | **2.92211E-07** | 0.09 | **4.57916E-06** |
| IP-10 | 0.16 | 0.57 | 0.74 | 0.99 | 0.51 | **0.02** | 0.08 | **0.0006** |

The statistical analysis was carried out using two-tailed t test and significant values are given in bold.

Abbr.: IL – interleukin, IFNγ – interferon gamma, TNFα – tumour necrosis factor alpha, GM-CSF – granulocyte macrophage colony stimulating factor, IP – interferon gamma-inducible protein.

Table S5.2: Statistical comparison of cytokine level within primary and secondary infection of different serotypes

| Cytokine | Primary infection | | | | | | Secondary infection | | | | | |
| --- | --- | --- | --- | --- | --- | --- | --- | --- | --- | --- | --- | --- |
|  | DENV1 vs. DENV2 | DENV1 vs. DENV3 | DENV1 vs. DENV4 | DENV2 vs. DENV3 | DENV2 vs. DENV4 | DENV3 vs. DENV4 | DENV1 vs. DENV2 | DENV1 vs. DENV3 | DENV1 vs. DENV4 | DENV2 vs. DENV3 | DENV2 vs. DENV4 | DENV3 vs. DENV4 |
| IL1β | 0.42 | 0.58 | 0.12 | 0.25 | 0.57 | 0.10 | 0.88 | 0.06 | 0.40 | **0.03** | 0.26 | 0.14 |
| IL2 | 0.44 | 0.07 | 0.63 | 0.14 | 0.66 | 0.10 | 0.66 | 0.09 | 0.70 | 0.09 | 0.16 | **0.03** |
| IL4 | 0.91 | 0.15 | 0.84 | 0.16 | 0.93 | 0.16 | 0.65 | 0.70 | 0.41 | 0.38 | 0.61 | 0.24 |
| IL6 | 0.14 | **0.02** | 0.39 | **0.002** | **0.03** | 0.08 | 0.64 | 0.90 | 0.96 | 0.55 | 0.35 | 0.91 |
| IL8 | 0.67 | 0.46 | 0.27 | 0.32 | 0.19 | 1.00 | 0.89 | 0.68 | 0.64 | 0.74 | 0.50 | 0.51 |
| IL10 | 0.54 | 0.31 | 0.25 | 0.54 | 0.43 | 0.99 | 0.38 | 0.27 | 0.30 | 0.63 | 0.75 | 0.77 |
| IL12 | 0.56 | 0.54 | 0.96 | 0.93 | 0.51 | 0.50 | 0.35 | 0.20 | 0.24 | 0.40 | 0.41 | 0.70 |
| IL17A | 0.56 | 0.24 | 0.31 | 0.39 | 0.72 | 0.51 | 0.35 | 0.41 | 0.20 | 0.84 | 0.37 | 0.32 |
| IFNγ | 0.70 | 0.59 | 0.35 | 0.74 | 0.51 | 0.96 | 0.09 | 0.13 | 0.95 | 0.58 | 0.13 | 0.11 |
| TNFα | 0.93 | 0.16 | 0.67 | 0.10 | 0.50 | 0.19 | 0.73 | 0.35 | **0.03** | 0.47 | **0.01** | 0.17 |
| GM-CSF | 0.26 | 0.58 | 0.12 | 0.99 | 0.80 | 0.92 | 0.96 | 0.95 | 0.55 | 0.97 | 0.40 | 0.72 |
| IP-10 | 0.36 | 0.17 | 0.15 | 0.45 | 0.54 | 0.75 | 0.31 | 0.13 | 0.24 | 0.31 | 0.85 | 0.33 |

The statistical analysis was carried out using two-tailed t test and significant values are given in bold.

Abbr.: IL – interleukin, IFNγ – interferon gamma, TNFα – tumour necrosis factor alpha, GM-CSF – granulocyte macrophage colony stimulating factor, IP – interferon gamma-inducible protein.

Table S5.3: Statistical comparison of cytokine level between primary and secondary infection of same serotypes

| Cytokine | D1 Primary Vs D1 Secondary | D2 Primary Vs D2 Secondary | D3 Primary Vs D3 Secondary | D4 Primary Vs D4 Secondary |
| --- | --- | --- | --- | --- |
| IL1β | 0.15 | 0.22 | 0.60 | 0.61 |
| IL2 | 0.38 | 0.99 | **0.02** | >0.05 |
| IL4 | 0.83 | 0.96 | 0.09 | 0.63 |
| IL6 | 0.47 | 0.06 | 0.69 | 0.52 |
| IL8 | 0.58 | 0.96 | 0.69 | **0.006** |
| IL10 | 0.87 | 0.16 | 0.31 | 0.12 |
| IL12 | 0.71 | **0.04** | 0.08 | **0.01** |
| IL17A | 0.26 | 0.98 | 0.60 | 0.09 |
| IFNγ | **0.002** | **0.03** | 0.64 | **0.0005** |
| TNFα | 0.30 | 0.32 | 0.09 | 0.40 |
| GM-CSF | **0.002** | **1.54953E-05** | 0.13 | **0.0001** |
| IP-10 | 0.16 | **0.01** | 0.09 | **0.0003** |

The statistical analysis was carried out using two-tailed t test and significant values are given in bold.

Abbr.: IL – interleukin, IFNγ – interferon gamma, TNFα – tumour necrosis factor alpha, GM-CSF – granulocyte macrophage colony stimulating factor, IP – interferon gamma-inducible protein.

Table S6: Cytokines expression among different serotypes with respect to disease severity i.e., DF, DHF and DSS

| Cytokine | Control (N=20) | DENV-1 DF (N=4) | DENV-2 DF (N=14) | DENV-4 DF (N=3) | DENV-1 DHF (N=5) | DENV-2 DHF (N=5) | DENV-3 DHF (N=4) | DENV-4 DHF (N=21) | DENV-1 DSS (N=5) | DENV-2 DSS (N=3) | DENV-3 DSS (N=3) | DENV-4 DSS (N=9) |
| --- | --- | --- | --- | --- | --- | --- | --- | --- | --- | --- | --- | --- |
| IL1β | 1.99±1.63 | 0.65±0.48 | 2.02±1.14 | 0.6±0.69 | 1.84±0.68 | 1.94±1.61 | 1±0.72 | 2±1.14 | 2.29±0.8 | 2.17±0.85 | 1.09±0.72 | 2.2±1.19 |
| IL2 | 2.58±2.12 | 3.83±0.59 | 2.69±0.99 | 4.7±1.67 | 2.3±1.86 | 3.04±2 | 2.9±2.39 | 2.63±1.11 | 2.8±1.22 | 3.47±1.5 | 2.57±2.08 | 2.43±1.51 |
| IL4 | 4.55±2.82 | 2.98±0.43 | 4.39±1.7 | 3.37±0.85 | 5.24±2.55 | 3.8±1.12 | 4.7±1.81 | 4.3±1.56 | 3.28±0.75 | 2.87±0.47 | 5.17±2.8 | 4.2±1.91 |
| IL6 | 22.85±11.15 | 30.2±7.41 | 29.84±8.53 | 26.47±4.75 | 21.4±5.97 | 34±9.85 | 20.62±3.58 | 27.96±8.19 | 31.38±1.67 | 29.73±6.53 | 25±6.84 | 19.76±5.68 |
| IL8 | 58.78±20.15 | 69.48±15.48 | 68.73±28.27 | 58.33±10.58 | 67.18±18.33 | 47.88±9.5 | 81.6±11.32 | 73.12±23.1 | 61.05±12.87 | 50.77±5.42 | 56.73±16.74 | 59.61±13.54 |
| IL10 | 25.25±16.01 | 49.38±23.64 | 39.32±12.7 | 22.07±0.46 | 35.64±14.67 | 33.46±7.92 | 32.72±9.38 | 35.28±12.96 | 40.88±14.73 | 20.13±1.03 | 30.13±12.01 | 32.14±9.66 |
| IL12 | 40.99±28.74 | 43.85±16.17 | 44.99±10.69 | 36±6.22 | 51.34±16.52 | 47.02±9.82 | 42.48±16.01 | 46.25±12.24 | 40.33±3.06 | 43.17±21.5 | 42.87±5.31 | 37.36±12.49 |
| IL17A | 3.08±2.91 | 3.1±1.49 | 3.26±1.91 | 2.57±0.58 | 3.08±1.81 | 3.82±2.08 | 3.85±1.43 | 4.04±2.04 | 5.58±3.15 | 3.9±2.98 | 4.5±1.97 | 2.01±1.03 |
| IFNγ | 15±5.25 | 31.13±9.48 | 30.36±12.58 | 20.23±7.64 | 28.1±12.5 | 33.2±12.81 | 25.53±5.27 | 32.24±12.1 | 40.15±22.87 | 38.03±7.84 | 39.3±14.69 | 23.93±9.85 |
| TNFα | 19.01±3.01 | 26.13±12.45 | 25.04±5.7 | 14.57±6.16 | 16.7±9.81 | 18.98±10.41 | 14.35±6.51 | 19.05±6.53 | 26.25±5.66 | 18.43±7.7 | 21.5±5.62 | 19.47±10.46 |
| GM-CSF | 2.46±1.79 | 2.7±1.49 | 4.43±2.48 | 3.8±1.32 | 3.86±1.98 | 3.94±1.44 | 5.13±3.29 | 3.91±1.73 | 4.65±3.2 | 6.37±0.45 | 4.1±2.31 | 5.16±2.51 |
| IP10 | 708.11±189.15 | 472.55±147 | 694.19±177.04 | 654.23±154.68 | 738.36±175.37 | 912.65±205.26 | 793.93±187.58 | 758.69±196.93 | 755.6±132.34 | 856.88±138.99 | 982.57±273.33 | 861.57±151.34 |

The cytokine expression was given in mean±SD.

Abbr.: IL – interleukin, IFNγ – interferon gamma, TNFα – tumour necrosis factor alpha, GM-CSF – granulocyte macrophage colony stimulating factor, IP – interferon gamma-inducible protein.

Table S6.1: Statistical comparison of cytokine level between control and different serotypes in disease severity (DF, DHF and DSS)

| Cytokine | DF | | | DHF | | | | DSS | | | |
| --- | --- | --- | --- | --- | --- | --- | --- | --- | --- | --- | --- |
|  | Control vs. DENV1 | Control vs. DENV2 | Control vs. DENV4 | Control vs. DENV1 | Control vs. DENV2 | Control vs. DENV3 | Control vs. DENV4 | Control vs. DENV1 | Control vs. DENV2 | Control vs. DENV3 | Control vs. DENV4 |
| IL-1β | **0.01** | 0.95 | **0.04** | 0.76 | 0.96 | 0.08 | 0.97 | 0.58 | 0.78 | 0.16 | 0.69 |
| IL-2 | **0.04** | 0.85 | 0.14 | 0.78 | 0.66 | 0.82 | 0.93 | 0.78 | 0.43 | 0.99 | 0.83 |
| IL-4 | **0.03** | 0.84 | 0.17 | 0.62 | 0.36 | 0.90 | 0.73 | 0.10 | **0.02** | 0.75 | 0.70 |
| IL-6 | 0.15 | **0.05** | 0.37 | 0.70 | 0.06 | 0.48 | 0.10 | **0.004** | 0.20 | 0.67 | 0.33 |
| IL-8 | 0.28 | 0.27 | 0.96 | 0.40 | 0.10 | **0.01** | **0.04** | 0.78 | 0.17 | 0.86 | 0.90 |
| IL-10 | 0.13 | **0.01** | 0.39 | 0.21 | 0.13 | 0.25 | **0.03** | 0.12 | 0.17 | 0.57 | 0.16 |
| IL-12 | 0.79 | 0.57 | 0.51 | 0.31 | 0.45 | 0.89 | 0.46 | 0.92 | 0.89 | 0.79 | 0.64 |
| IL-17A | 0.98 | 0.82 | 0.49 | 1.00 | 0.53 | 0.44 | 0.23 | 0.22 | 0.69 | 0.35 | 0.16 |
| IFNγ | **0.04** | **0.001** | 0.36 | 0.08 | **0.03** | **0.02** | **2.12132E-06** | 0.11 | **0.03** | 0.10 | **0.03** |
| TNFα | 0.34 | **0.002** | 0.34 | 0.63 | 1.00 | 0.25 | 0.98 | 0.08 | 0.91 | 0.53 | 0.90 |
| GM-CSF | 0.79 | **0.02** | 0.21 | 0.20 | 0.09 | 0.20 | **0.01** | 0.27 | **9.32864E-07** | 0.34 | **0.01** |
| IP-10 | **0.04** | 0.83 | 0.62 | 0.74 | 0.09 | 0.45 | 0.41 | 0.57 | 0.19 | 0.22 | **0.03** |

The statistical analysis was carried out using two-tailed t test and significant values are given in bold.

Abbr.: IL – interleukin, IFNγ – interferon gamma, TNFα – tumour necrosis factor alpha, GM-CSF – granulocyte macrophage colony stimulating factor, IP – interferon gamma-inducible protein.

Table S6.2: Statistical comparison of cytokine level in serotypes and disease severity (DF, DHF and DSS)

| Cytokine | DF | | | DHF | | | | | | DSS | | | | | |
| --- | --- | --- | --- | --- | --- | --- | --- | --- | --- | --- | --- | --- | --- | --- | --- |
|  | DENV1 vs. DENV2 | DENV1 vs. DENV4 | DENV2 vs. DENV4 | DENV1 vs. DENV2 | DENV1 vs. DENV3 | DENV1 vs. DENV4 | DENV2 vs. DENV3 | DENV2 vs. DENV4 | DENV3 vs. DENV4 | DENV1 vs. DENV2 | DENV1 vs. DENV3 | DENV1 vs. DENV4 | DENV2 vs. DENV3 | DENV2 vs. DENV4 | DENV3 vs. DENV4 |
| IL-1β | **0.004** | 0.92 | **0.04** | 0.90 | 0.12 | 0.69 | 0.29 | 0.94 | 0.06 | 0.85 | 0.09 | 0.87 | 0.17 | 0.96 | 0.10 |
| IL-2 | **0.02** | 0.46 | 0.16 | 0.56 | 0.70 | 0.72 | 0.93 | 0.68 | 0.84 | 0.57 | 0.87 | 0.66 | 0.58 | 0.93 | 0.93 |
| IL-4 | **0.01** | 0.52 | 0.17 | 0.30 | 0.72 | 0.47 | 0.43 | 0.43 | 0.70 | 0.42 | 0.36 | 0.24 | 0.29 | 0.62 | 0.62 |
| IL-6 | 0.94 | 0.46 | 0.39 | **0.05** | 0.82 | 0.08 | **0.04** | 0.26 | **0.02** | 0.71 | 0.25 | **0.0002** | 0.43 | 0.32 | 0.32 |
| IL-8 | 0.95 | 0.31 | 0.31 | 0.08 | 0.19 | 0.56 | **0.003** | **0.001** | 0.29 | 0.22 | 0.73 | 0.86 | 0.61 | 0.81 | 0.81 |
| IL-10 | 0.47 | 0.10 | **0.0002** | 0.78 | 0.73 | 0.96 | 0.90 | 0.70 | 0.66 | 0.07 | 0.34 | 0.34 | 0.29 | 0.81 | 0.81 |
| IL-12 | 0.90 | 0.42 | 0.11 | 0.63 | 0.44 | 0.54 | 0.64 | 0.88 | 0.68 | 0.84 | 0.51 | 0.52 | 0.98 | 0.31 | 0.31 |
| IL-17A | 0.86 | 0.55 | 0.27 | 0.57 | 0.50 | 0.33 | 0.98 | 0.84 | 0.83 | 0.51 | 0.60 | 0.11 | 0.79 | 0.15 | 0.15 |
| IFNγ | 0.90 | 0.15 | 0.13 | 0.54 | 0.69 | 0.53 | 0.27 | 0.88 | 0.10 | 0.87 | 0.95 | 0.25 | 0.90 | 0.20 | 0.20 |
| TNFα | 0.88 | 0.17 | 0.08 | 0.73 | 0.68 | 0.63 | 0.44 | 0.99 | 0.25 | 0.22 | 0.33 | 0.16 | 0.61 | 0.68 | 0.68 |
| GM-CSF | 0.12 | 0.35 | 0.56 | 0.94 | 0.53 | 0.96 | 0.54 | 0.97 | 0.52 | 0.36 | 0.80 | 0.79 | 0.23 | 0.54 | 0.54 |
| IP-10 | **0.05** | 0.19 | 0.72 | 0.19 | 0.66 | 0.83 | 0.40 | 0.18 | 0.75 | 0.38 | 0.29 | 0.25 | 0.53 | 0.53 | 0.53 |

The statistical analysis was carried out using two-tailed t test and significant values are given in bold.

Abbr.: IL – interleukin, IFNγ – interferon gamma, TNFα – tumour necrosis factor alpha, GM-CSF – granulocyte macrophage colony stimulating factor, IP – interferon gamma-inducible protein.

Table S6.3: Statistical comparison of cytokine level within serotypes with respect to disease severity (DF, DHF and DSS)

| Cytokine | DENV-1 | | | DENV-2 | | | DENV-3 | DENV-4 | | |
| --- | --- | --- | --- | --- | --- | --- | --- | --- | --- | --- |
|  | DF .vs DHF | DF .vs DSS | DHF .vs DSS | DF .vs DHF | DF .vs DSS | DHF .vs DSS | DHF .vs DSS | DF .vs DHF | DF .vs DSS | DHF .vs DSS |
| IL1β | **0.02** | **0.02** | 0.40 | 0.92 | 0.81 | 0.80 | 0.87 | **0.04** | **0.03** | 0.67 |
| IL2 | 0.15 | 0.20 | 0.64 | 0.72 | 0.47 | 0.74 | 0.85 | 0.16 | 0.12 | 0.73 |
| IL4 | 0.12 | 0.52 | 0.17 | 0.40 | **0.01** | 0.16 | 0.82 | 0.19 | 0.33 | 0.89 |
| IL6 | 0.10 | 0.78 | **0.02** | 0.43 | 0.98 | 0.49 | 0.39 | 0.67 | 0.11 | **0.005** |
| IL8 | 0.84 | 0.44 | 0.58 | **0.03** | **0.04** | 0.60 | 0.10 | 0.12 | 0.87 | 0.057 |
| IL10 | 0.36 | 0.57 | 0.61 | 0.26 | **0.0001** | **0.02** | 0.77 | **0.0001** | **0.01** | 0.47 |
| IL12 | 0.52 | 0.70 | 0.21 | 0.71 | 0.90 | 0.79 | 0.97 | 0.07 | 0.81 | 0.09 |
| IL17A | 0.99 | 0.22 | 0.22 | 0.62 | 0.75 | 0.97 | 0.66 | **0.02** | 0.29 | **0.001** |
| IFNγ | 0.69 | 0.51 | 0.39 | 0.68 | 0.24 | 0.53 | 0.24 | 0.09 | 0.53 | 0.06 |
| TNFα | 0.26 | 0.99 | 0.11 | 0.27 | 0.27 | 0.94 | 0.18 | 0.33 | 0.36 | 0.91 |
| GM-CSF | 0.35 | 0.33 | 0.68 | 0.61 | **0.02** | **0.02** | 0.65 | 0.90 | 0.27 | 0.20 |
| IP-10 | **0.04** | **0.03** | 0.87 | 0.08 | 0.16 | 0.66 | 0.37 | 0.37 | 0.13 | 0.14 |

The statistical analysis was carried out using two-tailed t test and significant values are given in bold.

Abbr.: IL – interleukin, IFNγ – interferon gamma, TNFα – tumour necrosis factor alpha, GM-CSF – granulocyte macrophage colony stimulating factor, IP – interferon gamma-inducible protein.

Table S7: Clinical presentation in DENV serotypes

| Serotype | DENV-1 (N=14) | | DENV-2 (N=22) | | DENV-3 (N=7) | | DENV-4 (N=33) | |
| --- | --- | --- | --- | --- | --- | --- | --- | --- |
| Dengue severity | DHF (n=5) | DSS (n=5) | DHF (n=5) | DSS (n=3) | DHF (n=4) | DSS (n=3) | DHF (n=21) | DSS (n=9) |
| Thrombocytopenia | 2 (14.3%) | 2 (14.3%) | 2 (9.1%) | 2 (9.1%) | 2 (28.6%) | 1 (14.3%) | 8 (24.2%) | 6 (18.2%) |
| Abdominal pain | 3 (21.4%) | 2 (14.3%) | 4 (18.2%) | 3 (13.6%) | 3 (42.9%) | 3 (42.9%) | 11 (33.3%) | 7 (21.2%) |
| Epistaxis | 1 (7.1%) | 3 (21.4%) | 0 | 0 | 0 | 0 | 0 | 1 (3.0%) |
| Serous effusion | 2 (14.3%) | 2 (14.3%) | 2 (9.1%) | 1 (4.5%) | 1 (14.3%) | 1 (14.3%) | 4 (12.1%) | 6 (18.2%) |
| Petechiae | 4 (28.6%) | 4 (28.6%) | 2 (9.1%) | 2 (9.1%) | 2 (28.6%) | 1 (14.3%) | 11 (33.3%) | 8 (24.2%) |
| Mucosal bleeding | 3 (21.4%) | 3 (21.4%) | 2 (9.1%) | 2 (9.1%) | 2 (28.6%) | 1 (14.3%) | 4 (12.1%) | 4 (12.1%) |
| Ascites | 1 (7.1%) | 1 (7.1%) | 2 (9.1%) | 2 (9.1%) | 1 (14.3%) | 0 | 3 (9.1%) | 4 (12.1%) |
| Hepatomegaly | 1 (7.1%) | 2 (14.3%) | 1 (4.5%) | 3 (13.6%) | 0 | 2 (28.6%) | 8 (24.2%) | 7 (21.2%) |
| Hypotension | 2 (14.3%) | 1 (7.1%) | 2 (9.1%) | 3 (13.6%) | 2 (28.6%) | 1 (14.3%) | 5 (15.2%) | 6 (18.2%) |
| Shock | 1 (7.1%) | 2 (14.3%) | 1 (4.5%) | 2 (9.1%) | 1 (14.3%) | 1 (14.3%) | 4 (12.1%) | 4 (12.1%) |
| Spontaneous bleeding | 1 (7.1%) | 1 (7.1%) | 0 | 0 | 0 | 1 (14.3%) | 2 (6.1%) | 3 (9.1%) |
| Circulatory failure | 0 | 1 (7.1%) | 1 (4.5%) | 1 (4.5%) | 1 (14.3%) | 0 | 0 | 2 (6.1%) |

Abbr: DF – dengue fever, DHF – dengue hemorrhagic fever, DSS – dengue shock syndrome, DENV – dengue virus
