# Supplementary figures and images for: Emergence of Dengue 4 as Dominant Serotype During 2017 Outbreak in South India and Associated Cytokine Expression Profile
Source: Front Cell Infect Microbiol. 2021 Aug 10;11:681937. doi: 10.3389/fcimb.2021.681937 (PMC8382982; doi:10.3389/fcimb.2021.681937)

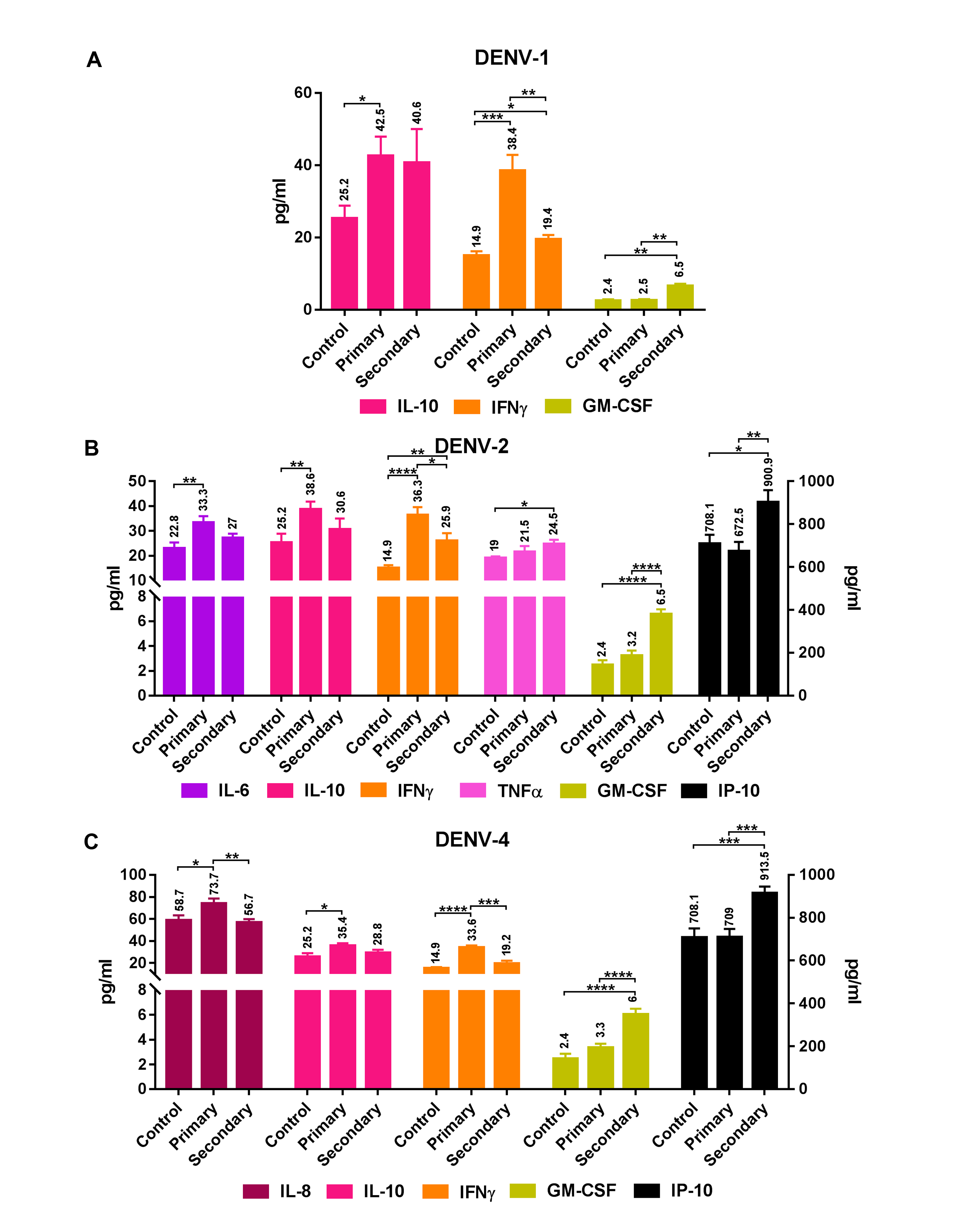

Supplement: Supplementary file 2 [file Image_1.tif]

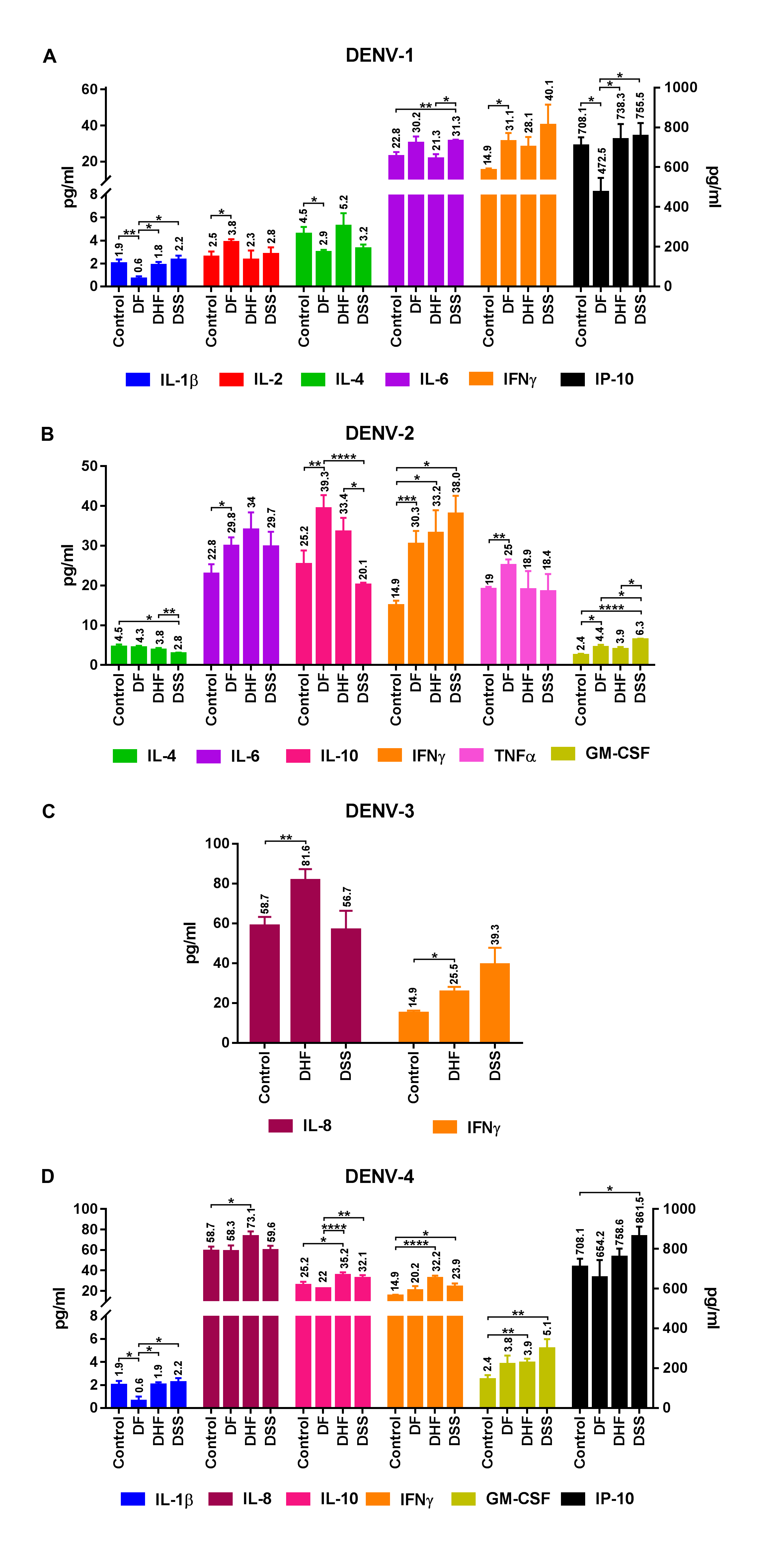

Supplement: Supplementary file 3 [file Image_2.tif]
